# Comparative transcriptomic insights into the evolution of vertebrate photoreceptor types

**DOI:** 10.1016/j.cub.2025.03.060

**Published:** 2025-04-17

**Authors:** Dario Tommasini, Takeshi Yoshimatsu, Teresa Puthussery, Tom Baden, Karthik Shekhar

**Affiliations:** 1 Helen Wills Neuroscience Institute, University of California, Berkeley, Berkeley, CA 94720, USA; 2 Department of Ophthalmology and Visual Sciences, Washington University in St. Louis School of Medicine, St. Louis, MO 63110, USA; 3 Vision Sciences Graduate Group, University of California, Berkeley, Berkeley, CA 94720, USA; 4 Herbert Wertheim School of Optometry and Vision Science, University of California, Berkeley, Berkeley, CA 94720, USA; 5 Center for Sensory Neuroscience and Computation, Sussex Neuroscience, School of Life Sciences, University of Sussex, Sussex, Brighton BN1 9QG, UK; 6 Department of Chemical and Biomolecular Engineering, University of California, Berkeley, Berkeley, CA 94720, USA; 7 Biological Systems and Engineering Division, Lawrence Berkeley National Laboratory, Berkeley, CA 94720, USA; 8 Lead contact

## Abstract

To explore the molecular similarities and potential evolutionary origins of vertebrate photoreceptor types, we analyzed single-cell and -nucleus transcriptomic atlases from six vertebrate species: zebrafish, chicken, lizard, opossum, ground squirrel, and human. Comparative analyses identified conserved transcriptional signatures for the five ancestral photoreceptor types: red, blue, green, and UV cones, as well as rods. We further identified and validated molecular markers of the principal and accessory members of the tetrapod double cone. Comparative transcriptomics suggests that the principal member originated from ancestral red cones, although the origin of the accessory member is less clear. The gene expression variation among cone types mirrors their spectral order (red → green → blue → UV). We find that rods are highly dissimilar to all cone types, suggesting that rods may have diverged prior to the spectral diversification of cones.

## In brief

Tommasini et al. show that rod and single-cone photoreceptors are orthologous across vertebrates, while non-eutherian tetrapods possess an additional transcriptionally distinct double cone. The double cone’s principal member likely evolved from the ancestral red cone, while the evolutionary origin of its accessory member is less clear.

## Graphical Abstract

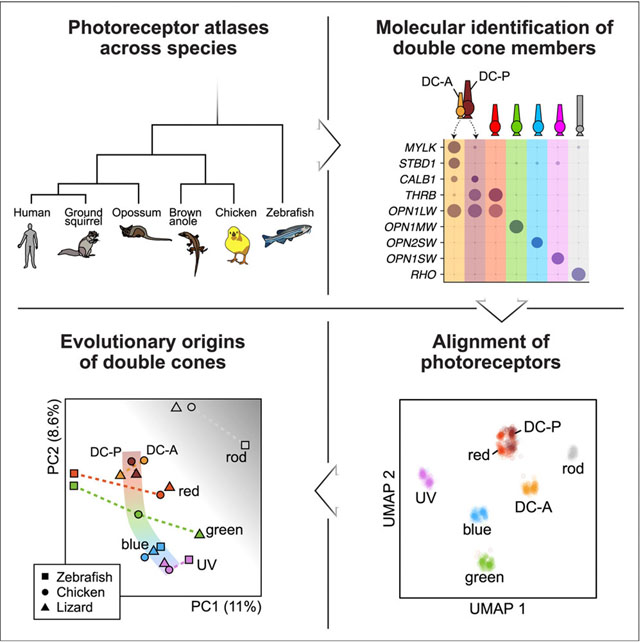

## INTRODUCTION

Eight types of ciliary photoreceptors have been identified across the retinas of extant vertebrates: rods, four types of single cones (red, green, blue, and UV), “secondary/green rods” of amphibians, and the two members of the tetrapod double cones (DCs).^1^ Rods and single cones are thought to be ubiquitous across vertebrates and, therefore, were likely present in the retina of the common vertebrate ancestor >500 million years ago (mya).^2–5^ In contrast, DCs and green rods likely emerged later, <390 mya, around the beginning of vertebrate life on land.^1^ In contrast to birds, reptiles, amphibians, and fish, eutherian mammals have only two cone types, which express the orthologs of the long-wave-sensitive (LWS) and short-wave-sensitive (SWS) opsins, suggesting that these types may be orthologous to the ancestral red and UV cones, respectively.^1,5^ However, the evolutionary relationships of vertebrate photoreceptors, from fish to birds and humans, remain incompletely understood.

The tetrapod DC is so called because it is composed of two tightly associated photoreceptors, the principal (DC-P) and the accessory (DC-A).^6,7^ DCs can be quite numerous, comprising over 40% of the cones in some avian retinas.^8^ Like ancestral red cones, DCs express the LWS opsin and are, therefore, expected to be broadly tuned for long wavelengths.^9,10^ Their large size and absence in the fovea of some raptorial birds suggest they may support fast achromatic processing.^11,12^ However, to our knowledge, outside of rare recordings in salamanders^9,10^ and turtles,^13^ direct functional insights into the physiology of tetrapod DCs remain outstanding.

**Figure 1.**
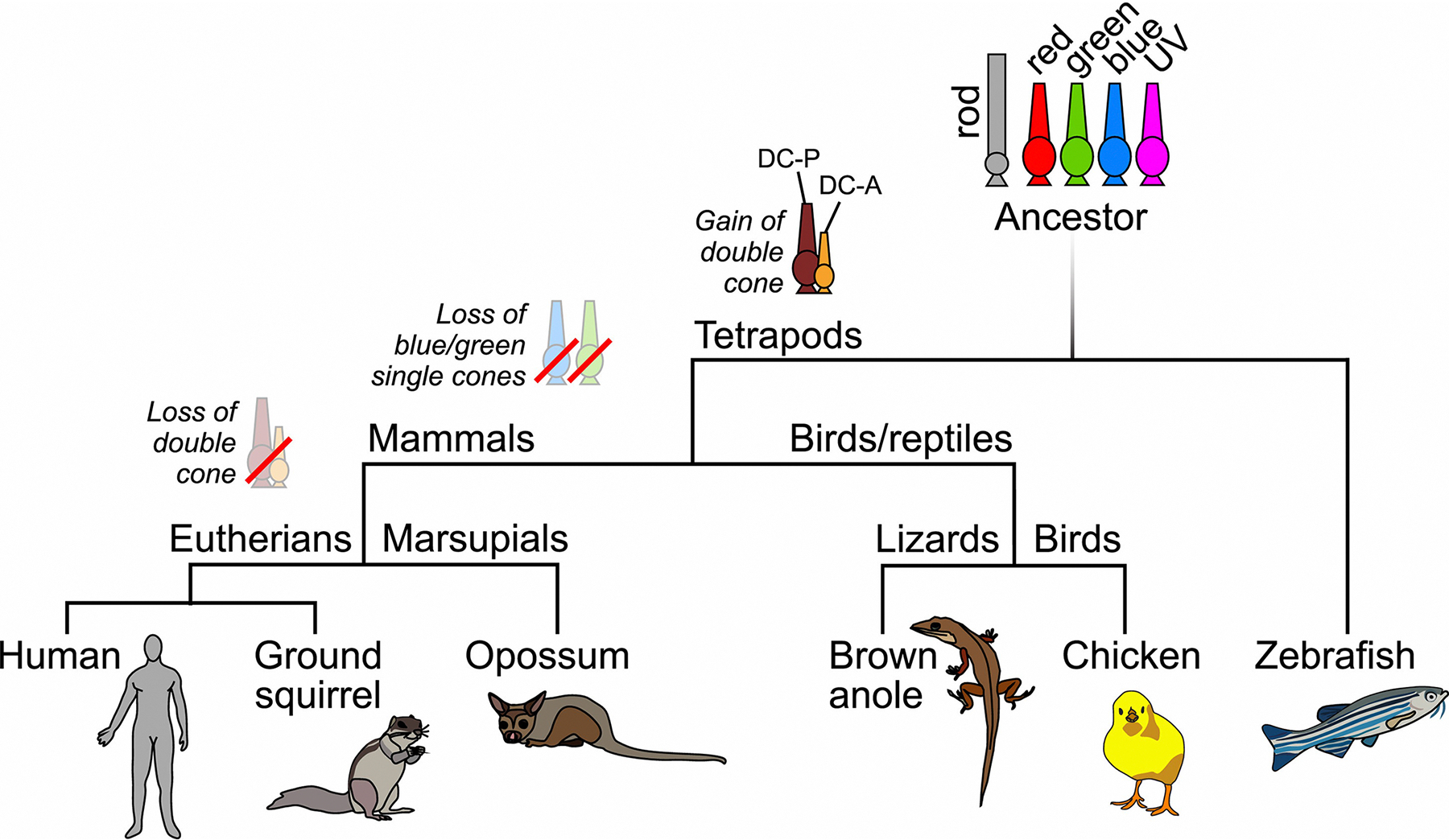
Phylogenetic tree showing the putative evolutionary history of photoreceptor diversity in the six studied vertebrates The vertebrate ancestor likely had rods and the four types of single cones. Then, double cones (DCs) were gained in tetrapods, followed by the loss of green and blue cones in mammals and DCs in eutherians.^1,5^ Branch lengths are not to scale.

The presence of an “extra” pair of DCs in birds, reptiles, amphibians, monotremes, and marsupials, but not in fish and other mammals, suggests that they arose in the common ancestor of tetrapods and were later lost in eutherian mammals.^1,5^ Importantly, the tetrapod DC exists in parallel to ancestral single cones, and is therefore distinct from anatomical pairs of red/green single cones of fish, which are also sometimes referred to as double or twin cones^14,15^ (see discussion). Although their evolutionary origin is debated, phylogeny suggests that DCs evolved from ancestral vertebrate single cones (Figure 1). One possibility—based on morphological similarity to pairs of red and green cones in fish retinas^14,16^—is that DC-P evolved from the ancestral red cone and DC-A evolved from the ancestral green cone.^1^ Alternatively, because both DC-P and DC-A express the LWS opsin, it has been suggested that both DC members may have evolved from the ancestral red cone.^9,11^ A third hypothesis—supported by the recent observation that the candelabra-shaped horizontal cell in chicken wires onto DC-A and blue cones^17^—is that DC-A is related to blue single cones. An independent study parallel to the present work supports this third scenario.^18^

## RESULTS

### Comparative analysis of photoreceptor atlases

**Figure 2.**
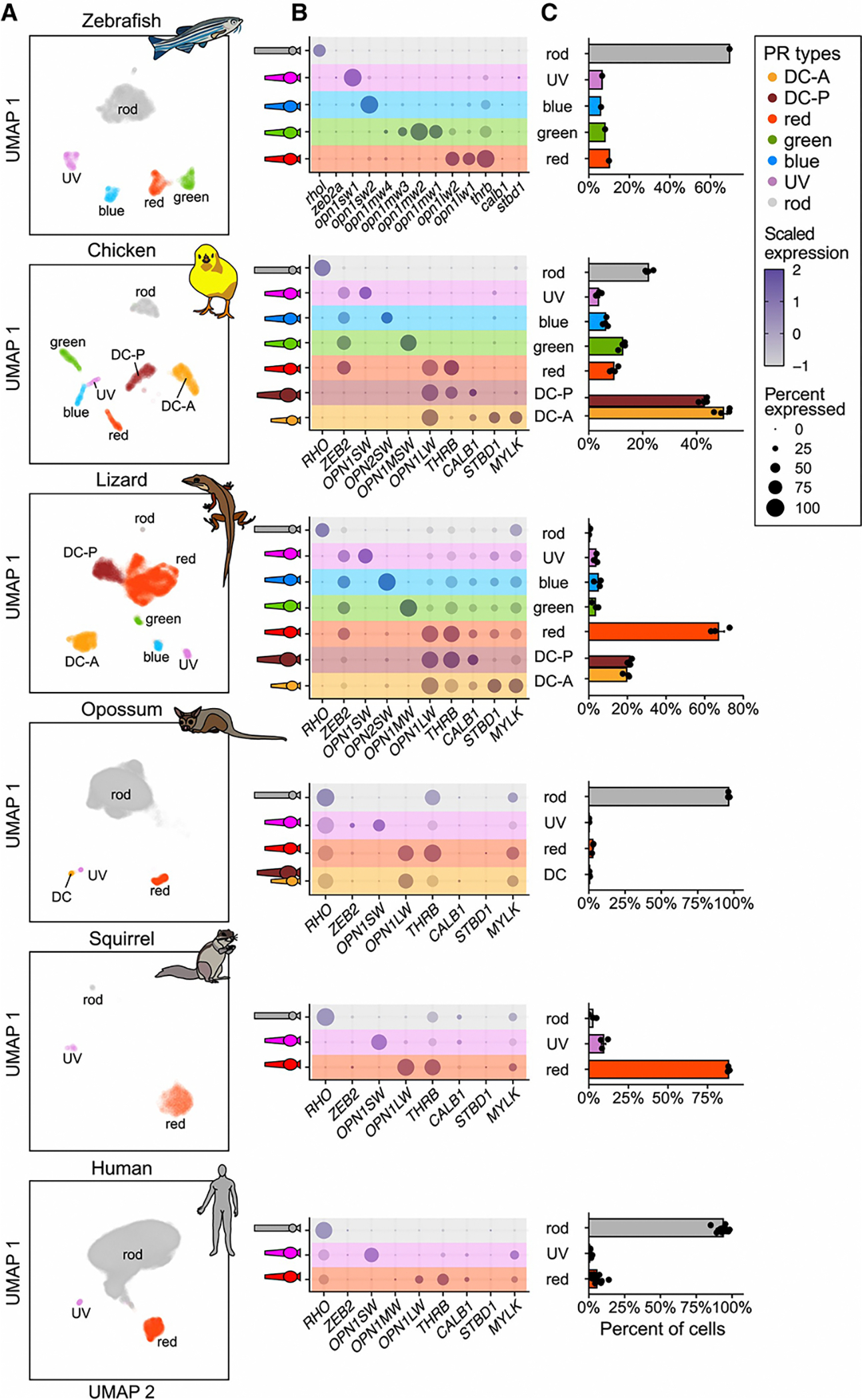
Single-cell atlases of photoreceptors across six vertebrate species (A) 2D uniform manifold approximation projection (UMAP)^22^ embeddings of single-cell atlases. Each subpanel (row) represents data from a different species. Colors correspond to photoreceptor types (see legend, right). (B) Dot plots showing expression of marker genes corresponding to cell types in (A). Color shows average expression scaled for each gene across photoreceptor types. Circle size corresponds to the percentage of cells in the cluster that express that gene (legend, right). (C) Relative proportion of photoreceptor types. Points correspond to biological replicates. The original chicken atlas had a third cluster composed of DC-P and DC-A doublets, which is not shown here (Figure S1). Note that the proportions consider DCs as a single photoreceptor type and were computed by averaging DC-A and DC-P numbers. Bars represent the mean and error bars represent ± 1 SD. See also Figure S1 and Table S1.

We hypothesized that similarity in gene expression might reveal the evolutionary relationships between vertebrate ciliary photoreceptor types. We therefore analyzed published single-cell (sc) and single-nucleus (sn) RNA-sequencing (RNA-seq) atlases of rods and cones from six vertebrate species: zebrafish (*D. rerio*),^19^ chicken (*G. gallus*),^20^ brown anole lizard (*A. sagrei*),^21^ opossum (*M. domestica*),^21^ thirteen-lined ground squirrel (*I. tridecemlineatus*),^21^ and human (*H. sapiens*)^21^ (Figure 2). By applying dimensionality reduction and clustering to each species atlas, we identified transcriptomic clusters corresponding to rods, single cones, and putative DCs (Figure 2A; Table S1). As expected, zebrafish, chicken, and lizard contained rods and the full complement of single cones (red, green, blue, and UV), whereas opossum, squirrel, and human contained rods and only two types of single cones, which correspond to ancestral red and UV cones. We note that mammalian cones are annotated based on their presumed ancestry rather than spectral sensitivity^5^ (STAR Methods). For instance, human blue cones express a blue-shifted variant of the SWS1 opsin and are likely derived from the ancestral UV cone, so they were annotated as UV cones.^5^

Beyond rods and single cones, we found two additional clusters in chicken and lizard representing putative DC members (Figures 2A and S1). These two clusters expressed *OPN1LW*, the gene encoding the LWS opsin, but unlike single cones in chicken and lizard, they did not express *ZEB2* (Figure 2B). In addition, these clusters were present in approximately 1:1 stoichiometric ratio in both species (Figure 2C). One of these clusters was enriched for the red cone marker *THRB*,^23,24^ whereas the other selectively expressed *STBD1* and *MYLK* (Figure 2B). Given that DC-A exhibits an enlarged glycogen-containing organelle (paraboloid) and *STBD1* is associated with glycogen metabolism,^25^ we hypothesized that the *OPN1LW*^+^ *THRB*^+^ *STBD1*^−^ *MYLK*^−^ cluster represents DC-P and the *OPN1LW*^+^ *THRB*^−^ *STBD1*^+^ *MYLK*^+^ cluster represents DC-A (STAR Methods) and validated them in tissue (see below). In opossum, we identified a cluster separate from the red cone cluster that was *OPN1LW*^+^ *ZEB2*^−^ *THRB*^low^, likely representing DCs, but there were not enough cells to resolve DC-P and DC-A separately (Figure 2B). As expected, zebrafish and the two eutherian mammals (squirrel and human) each contained a single *OPN1LW*^+^ cluster corresponding to ancestral red single cones. The overall taxonomy of tetrapod DCs with respect to single cones shown here is consistent with an independent study of chicken and green anole photoreceptor transcriptomes.^18^ Taken together, these results support the evolutionary scenario that a new DC, distinct from all cone types found in fish, arose with or after the emergence of vertebrate life on land and was later lost in eutherian mammals.^1^

The relative abundances of photoreceptor types within each species were consistent across biological replicates and were comparable with previous reports^8,26–28^ (Figures 2C, S1E, and S1F). Rod frequencies exhibited the highest variation, being >90% in the nocturnal opossum and <1% (but not absent) in the diurnal lizard. DCs were the most prevalent photoreceptor type in chicken (~45%) and the second-most prevalent type in lizard (~20%) but were quite rare in opossum (<1%).

### Transcriptional correspondence and orthology among ancestral photoreceptor types

**Figure 3.**
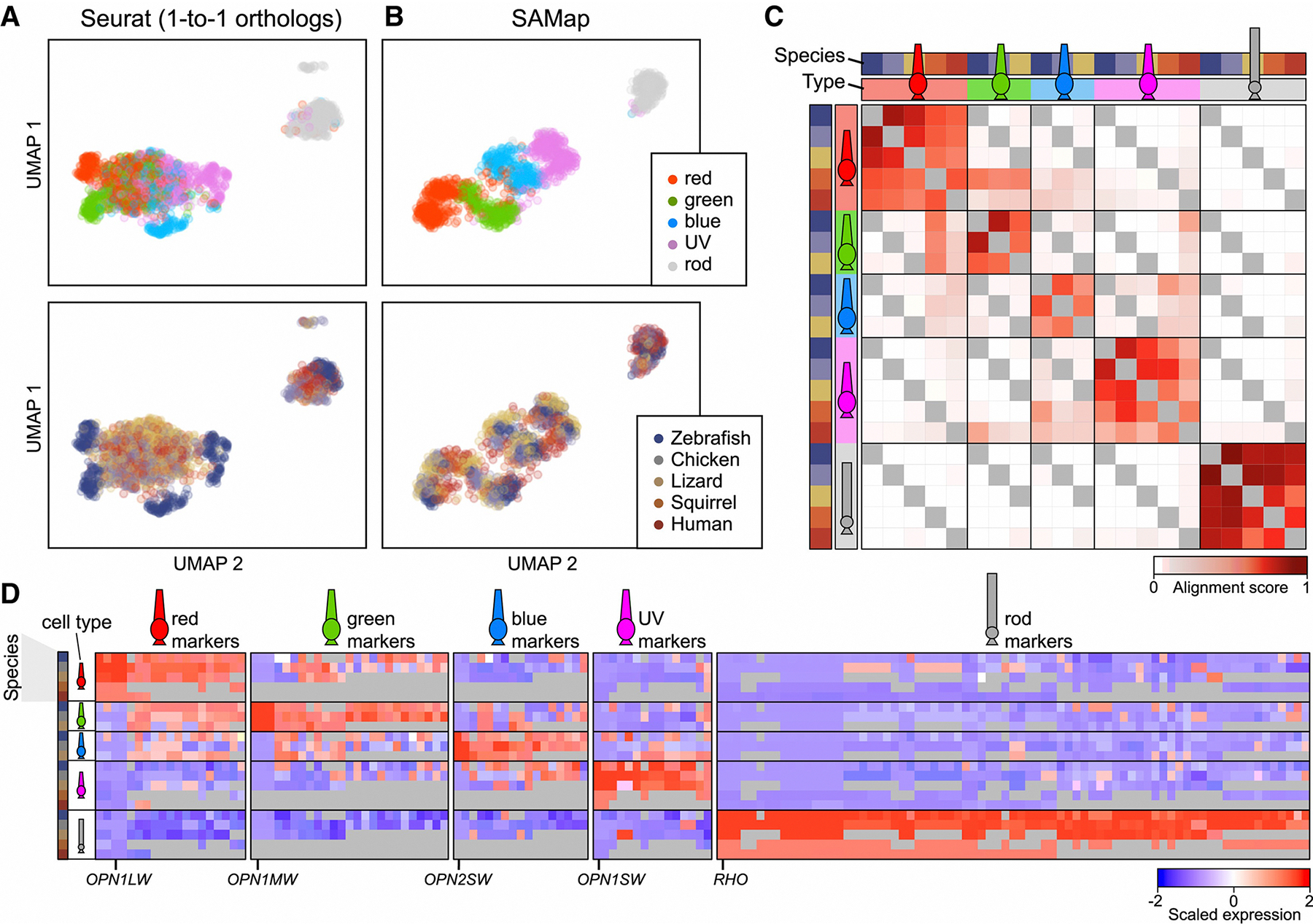
Cross-species integration of ancestral photoreceptors (A) 2D visualization of single-cone types and rods from six species integrated using Seurat v4,^29^ resulting in the intermixing of cone types. (B) Same as (A) but with integration performed using SAMap,^30^ which preserves cone type identity. (C) Heatmap of SAMap alignment scores for photoreceptor types. Alignment scores represent averaged similarity in the k-nearest-neighbor graph between two clusters.^30^ Evolutionarily related photoreceptors have high alignment scores. Values shown are means from 50 experimental runs with different subsamples of cells (STAR Methods). (D) Heatmap of scaled gene expression showing the top conserved transcriptional signatures of ancestral photoreceptor types. Because genes can have multiple homologs, each column represents a unique combination of homologous genes across species. Gray values indicate that no gene homolog was found for that species. Some opsin genes are highlighted; for all gene names, see Figure S2. See also Figures S2 and S3.

We first asked whether we could recover the long-suspected evolutionary relationships of the ancestral photoreceptors (single cones and rods). We began with an integration approach that relies on 1:1 orthologous genes,^21,29^ but this method failed to fully separate the different cone types (Figure 3A). We then applied an alternative approach, SAMap, which incorporates complex gene homology relationships and iterative refinement.^30^ SAMap recovered the expected photoreceptor homologies among zebrafish, chicken, and lizard photoreceptors, each of which have the full complement of single cones and rods. Furthermore, the mammalian photoreceptor types co-clustered with their non-mammalian counterparts (Figures 3B and 3C). The conserved transcriptional signatures for the five ancestral photoreceptors included their respective opsins—*RHO* in rods, *OPN1LW* in red cones, *OPN1MW* in green cones, *OPN2SW* in blue cones, and *OPN1SW* in UV cones—as well as known photoreceptor genes such as *GNGT1*, *PDE6B*, *PDE6H*, and *THRB*,^31,32^ demonstrating SAMap’s ability to correctly identify orthologous genes (Figure 3D). This transcriptional homology is supported by many genes (Figure S2) and is robust even after the exclusion of opsins (Figures S3A–S3D). Together, these data support the orthology of rods and the four single-cone types across vertebrates, and the markers provided here will be useful for classifying cone types in other species.

### Molecular identification of DC-A and DC-P

**Figure 4.**
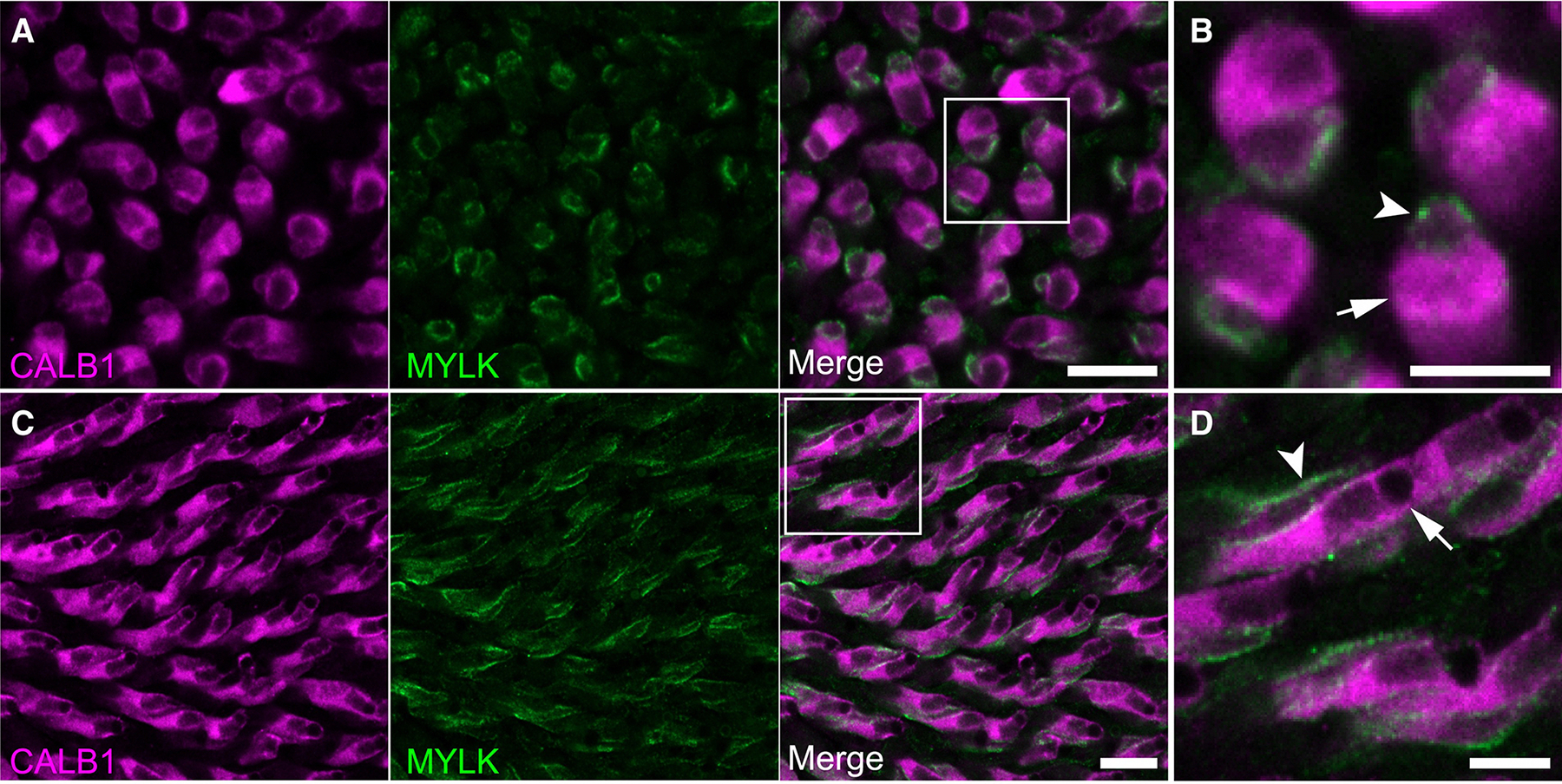
Molecular identification DCs (A) Confocal micrograph of a flat-mounted chicken retina (aged P7) showing DC inner segments immunolabeled for CALB1 and MYLK. (B) Enlargement of the boxed region in (A), showing the CALB1^+^MYLK^+^ DC-A (arrowhead) and the CALB1^+^MYLK^−^ DC-P (arrow). (C) Like (A) but in a different region where DCs are tilted to the right. (D) Enlargement of the boxed region in (C), showing the CALB1^+^MYLK^+^ DC-A (arrowhead) and the CALB1^+^MYLK^−^ DC-P (arrow pointing to oil droplet). Scale bars,10 μm (A and C) and 5 μm (B and D). See also Figure S4.

We next analyzed the transcriptional profiles of DC clusters in chicken. Both DC-A and DC-P express *CALB1* (Figure 2B), which encodes calbindin, a well-established marker for avian DCs.^33–35^ However, *MYLK* expression distinguishes these populations, as it is present only in the putative DC-A cluster (Figure 2B). To validate these cluster assignments, we immunolabeled CALB1 and MYLK proteins in flat-mounted chicken retinas and imaged the photoreceptor inner segments *en face*. In this orientation, DCs display a characteristic “figure eight” shape, with DC-A being notably smaller than DC-P (Figure 4A).^35^ Immunostaining confirmed our transcriptomic findings: CALB1 protein was detected in both DC members, whereas MYLK was exclusively present in DC-A (Figure 4B, arrowhead). DC-P could also be readily identified by its distinctive oil droplet (Figures 4C and 4D, arrow), which is much smaller and often not visible in DC-A.^8,36,37^ These observations also confirmed that MYLK localization was restricted to DC-A (Figure 4D, arrowhead). Together, these results confirm that chicken DC-A is *CALB1*^+^*MYLK*^+^, whereas DC-P is *CALB1*^+^*MYLK*^−^. Additionally, we performed fluorescence *in situ* hybridization chain reaction (HCR)^38^ in chicken retinal sections to detect *OPN1LW*, *CALB1*, and *STBD1* (another marker of DC-A). We found that a portion of *CALB1*^+^ photoreceptors co-express *STBD1* but almost all *STBD1*^+^ cells co-express *CALB1* (Figures S4A–S4D), consistent with DC-A being *CALB1*^+^*STBD1*^+^ (Figure 2B). We also performed *in situ* HCR in green anole lizard retinal sections to detect *OPN1LW* and *STBD1* (Figures S4E–S4H). In both chicken and lizard, the relative abundances of *OPN1LW*^+^ and *STBD1*^+^ cells matched our observations from sc/snRNA-seq (Figure 2C).

### Comparative transcriptomics of DCs and single cones

**Figure 5.**
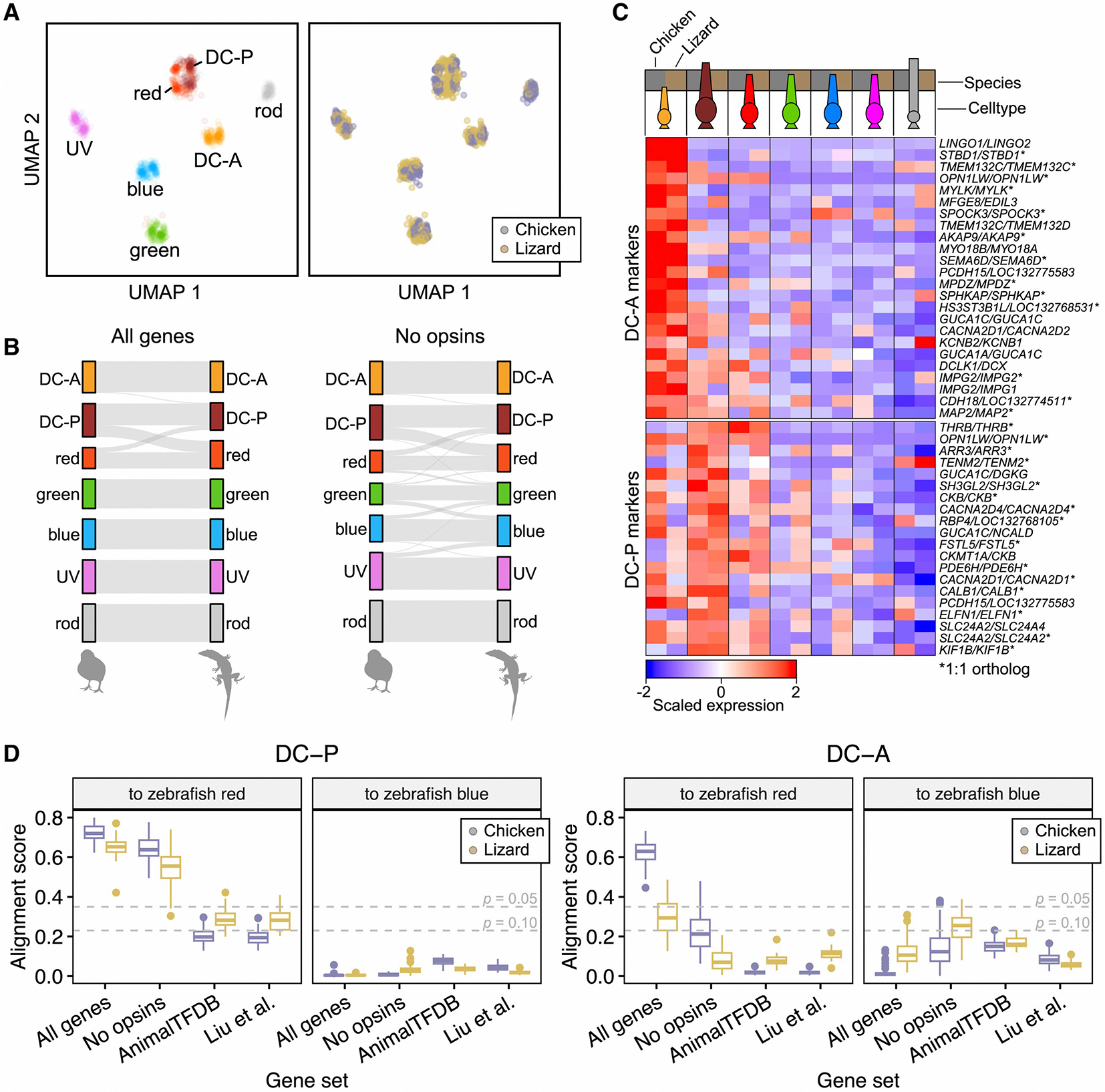
Comparative analysis of DCs (A) 2D embedding of the SAMap integration between chicken and lizard photoreceptors, including DC members. (B) Sankey diagram showing SAMap alignment between the photoreceptor types in chicken and lizard. Left panel shows the results of integration using all genes, whereas the right panel shows the results of integration after removal of opsin genes. The visualization is based on average alignment scores across 50 experiments with different subsamples of cells. (C) Top conserved genes of DC-A and DC-P, shown as a heatmap of scaled gene expression. 1:1 orthologs (as determined by reciprocal BLAST) are shown with an asterisk. (D) Box and whisker plots showing SAMap alignment scores to zebrafish red cone and zebrafish blue cone across 50 experiments. Left panels show DC-A scores whereas right panels show DC-P scores. In each panel, the dashed horizontal lines indicate the 0.05 and 0.10 significance level estimated from the five-species integration of ancestral photoreceptors (Figure 3C; STAR Methods). See also Figures S3, S5, and S6 and Data S1.

To explore the evolutionary conservation of DCs between chicken and lizard, we compared the transcriptional profiles of photoreceptors across these species using SAMap. DC-A and DC-P cells from each species clustered with their respective counterparts (Figure 5A). Although DC-A formed a distinct cluster, DC-P showed a tendency to cluster proximal to red cones (Figure 5A). SAMap alignment scores confirmed strong homology between species for both DC members (chicken DC-P/lizard DC-P, 0.52 ± 0.05; chicken DC-A/lizard DC-A, 0.76 ± 0.022), with a weaker, albeit consistently significant, alignment between DC-P and red cones (chicken DC-P/lizard red, 0.28 ± 0.07; chicken red/lizard DC-P, 0.1 ± 0.054) (Figure 5B, left). Notably, these alignment patterns persisted even after removing opsin genes from the analysis (Figure 5B, right). DC-A showed a highly distinctive transcriptional profile, selectively expressing *LINGO1*, *STBD1*, *MYLK*, *SEMA6D*, *MYO18B*, and *MPDZ*. DC-P’s profile, though less distinct, was characterized by elevated expression of *THRB*, *CALB1*, *KIF1B*, *ELFN1*, and *FSTL5* (Figure 5C).

To investigate the DC’s evolutionary origins, we compared chicken and lizard photoreceptors to the ancestral repertoire in zebrafish. Initial whole-transcriptome analyses suggested both DC-P and DC-A aligned best with zebrafish red cones (Figure 5D), although the DC-A alignment was weaker and DC-A has a greater number of differentially expressed genes (DEGs) (Figure S5). *Prima facie*, these patterns suggest that the DC-P and DC-A arose from ancestral red cones. In a parallel study, however, Liu et al.^18^ propose that cell-fate-determining transcription factors (TFs) are more reliable markers for evolutionary comparisons. Their analysis retains a red cone origin for DC-P but proposes a blue cone origin for DC-A. To explore this alternative perspective, we conducted additional SAMap analyses (1) excluding visual opsin genes, (2) restricting features to ~1,500 TFs from AnimalTFDB 3.0,^39^ and (3) restricting to ~100 highly variable TFs from Liu et al.^18^ (STAR Methods). The preferential alignment of DC-P to zebrafish red cones was largely insensitive to these variations (Figure 5D, left). However, removal of opsin genes and restriction to TFs increased the alignment of DC-A to blue cones, although the overall alignment scores remained weak (Figure 5D, right, and Figures S3E–S3H). Analysis of TF expression patterns revealed TFs shared between DC-A and blue cones (*SKOR1* in chicken and lizard; *MYO3B* and *HIVEP3* in chicken), as well as DC-A and red cones (*ISL2* in chicken and lizard; *RXRG* and *ARID3B* in chicken) (Figure 7B).

Overall, our analyses suggest that DC-P likely evolved from an ancestral red cone, but the evolutionary origin of DC-A is less clear due to its unique transcriptional profile and variable alignment patterns.

### Cone gene expression mirrors their spectral order

**Figure 6.**
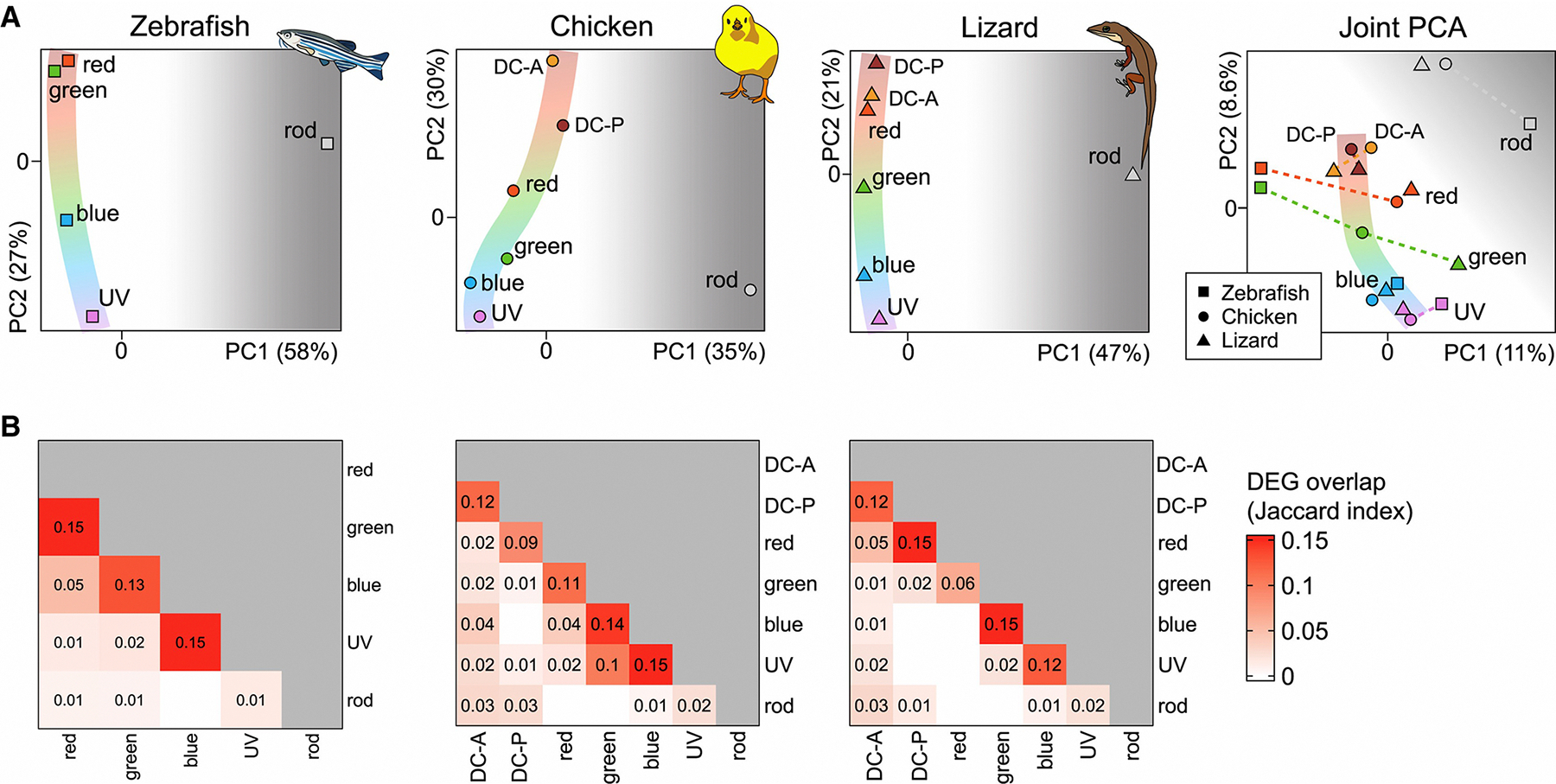
Transcriptomic variation among photoreceptors mirrors their spectral relationships (A) PCA embedding of photoreceptors in zebrafish, chicken, and lizard separately and in the joint expression space derived from SAMap. To reduce species-specific noise in the joint expression space, we binarized the scaled expression values as 0 (not expressed) or 1 (expressed) (STAR Methods). Cone types lie along the second principal component mirroring their spectral arrangement. (B) Heatmaps showing the overlap (Jaccard index) for markers in zebrafish (left), chicken (middle), and lizard (right). Positive markers shown here had a Benjamini-Hochberg-adjusted *p* < 0.01 and log_2_ fold change > 0.5, although other values produced similar results. Notice that in chicken and lizard, the cone types follow the similarity pattern DC-A → DC-P → red → green → blue → UV. Rods do not seem to participate in this pattern. See also Figure S7.

Across zebrafish, chicken, and lizard, we found that photoreceptors with similar spectral sensitivities share more similar gene expression patterns: blue cones show highest transcriptional similarity to UV and green cones, whereas green cones are most similar to blue and red cones (Figure S5). Principal-component analysis (PCA) revealed that the second principal component (PC2) consistently reflects the spectral ordering of cone types (Figure 6A). This pattern was reproducible in two independent zebrafish datasets^19,32^ (Figures S7A and S7B). Notably, both members of the DCs (DC-P and DC-A) positioned near red cones and extended into the “infrared” region of PC2 (Figure 6A). This positioning remained stable whether we included or excluded opsin genes or restricted the analysis to TFs (Figures S7C–S7F). In chicken, we identified key genes driving this spectral organization. Genes associated with longer wavelength sensitivity (positive PC2 loadings) included *ARR3*, *AKAP9*, *EIRCH1*, *RXRG*, and *SORCS2*. Conversely, genes associated with shorter wavelength sensitivity (negative PC2 loadings) included *PLEKHA2*, *NTNG1*, *NFIB*, and *NEBL* (Figure S7G).

### Rods are equally dissimilar to all cones

**Figure 7.**
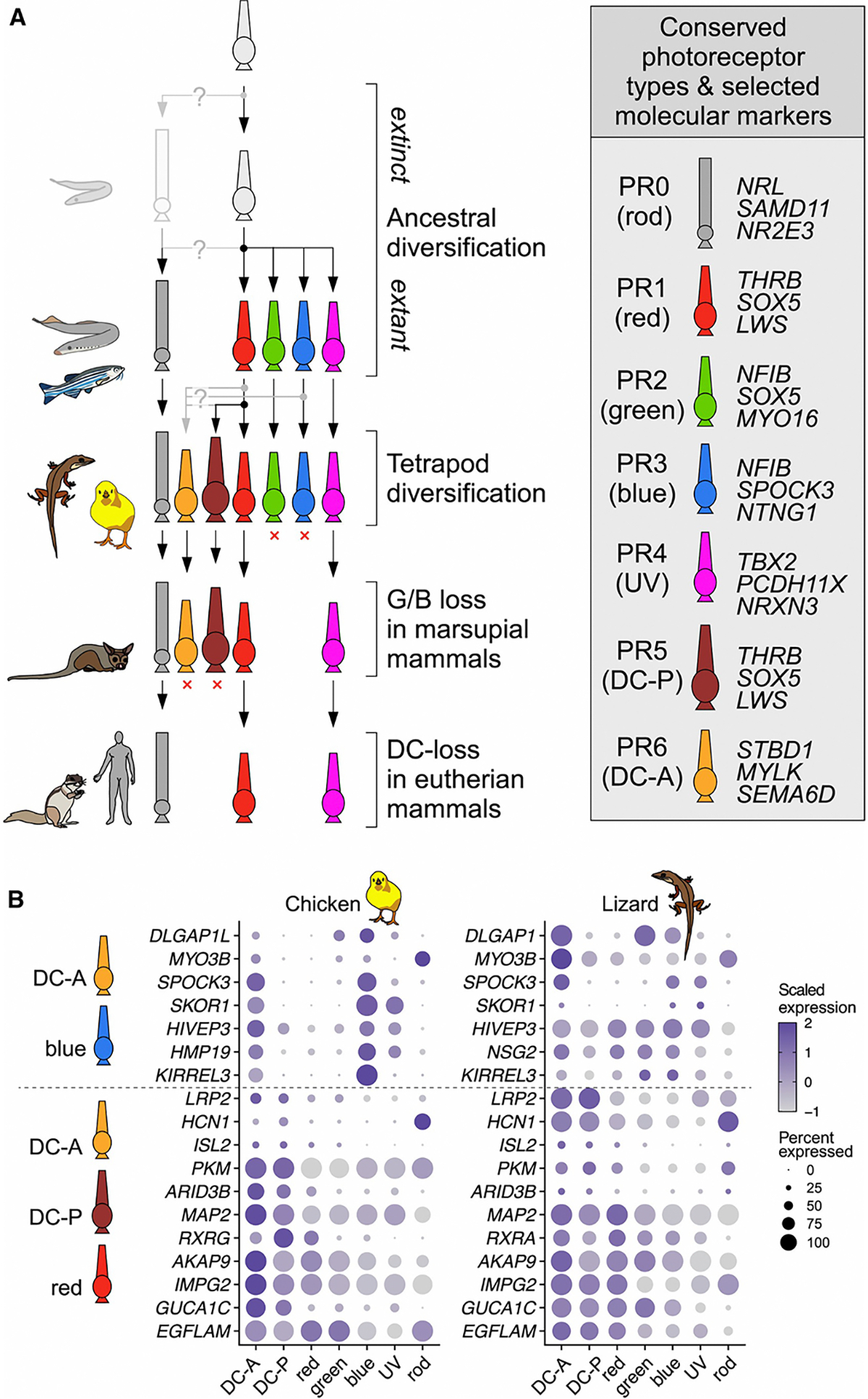
Putative sequence of photoreceptor evolution in vertebrates based on transcriptomic relationships Rods and cones diverged, followed by the spectral diversification of cones. This is followed by the emergence of DCs in tetrapods. DC-P likely evolved from red cones, whereas DC-A may have evolved from red or blue cones. Finally, DCs and blue and green cones were lost in eutherian mammals. The right panel highlights some conserved markers identified by SAMap (Figures 3D and S2). Many of these genes have been shown to be functionally relevant in their respective photoreceptor type (*NRL*,^43^ *THRB*,^46^ *TBX2*,^47,48^ and *NRXN3*^49^). (B) Dot plot summarizing the top genes driving the similarity between DC-A/DC-P/red and DC-A/blue cones. Genes presented here were compiled from the SAMap analysis (Figure 5D), the TF analysis (Figure S6), and DEG overlap analysis (Figure 6B).

Rods have long been speculated to have evolved from cones.^2,40^ The protein sequence similarity between rhodopsin and the green-sensitive opsin has been used as evidence to suggest that rods evolved from green cones.^2,41,42^ Other molecular signatures hint at a UV cone origin^43,44^ and the joint presence of red-cone-like and rod-like photoreceptors in the pineal organ of lower vertebrates, but the putative absence of green/blue/UV cones hints at a shared ancestry with red cones.^45^ As an attempt to distinguish between these scenarios, we examined the transcriptional similarity of rods and different cone types. We found that rods are highly dissimilar to all cone types and have no consistent affinity toward any cone type across zebrafish, chicken, and lizard based on DEGs (Figure S5). Additionally, in PCA space, the principal component capturing the highest variance (PC1) always separates rods and cones (Figures 6A and S7C–S7F). SAMap alignment scores with rods are high (>0.6), but alignment scores between rods and cone types are low (<0.1) and showed no consistent bias (Figures 3C and S3). Finally, the most common TF expression patterns in chicken and lizard photoreceptors are rod-specific and cone-specific TFs (Figures S6A and S6B). Ultimately, these findings suggest that rods and cones diverged before the spectral diversification of cone types or that rods have diverged beyond recognition from their cone-type predecessor (Figure 7A).

## DISCUSSION

Our integration of single-cell atlases across six vertebrate species shows the molecular conservation of photoreceptors and suggests potential evolutionary origins of DCs in tetrapods. Our main contributions are as follows:

### Transcriptomic atlases of photoreceptors

Through clustering analyses, we identified rods and single cones in each species and pinpointed clusters corresponding to DCs. In chicken and lizard, we resolved both DC-P and DC-A, though their lower abundance in opossum prevented such resolution. Although cross-species orthology among photoreceptor types has been long posited based on opsin expression alone, our whole-transcriptome analysis supports these relationships and provides new molecular markers for identifying photoreceptors in other species (Figures 3D, 5C, and S2). Having markers in addition to the opsins is particularly valuable, as focusing on opsin expression alone can sometimes mislead ancestry inference; for instance, ancestral red cones in the ventral retina of mice can co-express the SWS1 opsin,^50,51^ and zebrafish green cones can co-express the LWS opsin (data not shown).^32^

The abundance of different photoreceptor types varies markedly across species. DCs are most abundant in chicken, less common in lizard, and rare in opossum (Figure 2C). All six species show higher proportions of red and green cones compared with blue and UV cones. Notably, we discovered a small population of rods in anole lizards, contrary to the traditional view that diurnal reptiles lack rods.^52,53^ Indeed, even closely related species from the same group inhabiting distinct ecological niches may exhibit variations in the general patterns identified here,^50^ as we have recently seen while comparing diurnal vs. nocturnal rodents.^54^

### Patterns of gene expression in tetrapod DCs

We identified distinct molecular signatures that differentiate DC-A and DC-P from each other and from single cones in both chicken and lizard (Figures 5C and S5; Data S1). DC-A expresses a unique set of genes involved in three major functional categories: calcium-dependent cell-cell adhesion (*PCDH15* and *CDH18*), protein kinase A signaling (*SPHKAP* and *AKAP9*), and muscle-related proteins (*MYLK*, *MYO18A/B*, *MAP2*, and *STBD1*). The expression of muscle-related genes suggests either high metabolic activity or extensive intracellular trafficking needs for necessary molecules (e.g., retinal). Particularly intriguing is *STBD1*, a gene enriched in DC-A (Figure 2B). The product of this gene co-localizes with glycogen stores and is thought to anchor glycogen to membranes,^25^ and it may be linked to the DC-A’s enlarged paraboloid.^34,36,53^ Taken together, the molecular markers identified here not only provide tools for experimental manipulation but also offer insights into the specialized functions of DCs.

### Evolutionary origin of DC-A: Red or blue?

Although our analyses and a parallel study^18^ agree that DC-P likely evolved from ancestral red single cones, DC-A’s origins are more complex. When examining whole-cell transcriptomes, DC-A shows the strongest similarity first to DC-P and then to red single cones. However, when focusing specifically on TFs, DC-A appears more closely related to ancestral blue cones.^18^ This apparent contradiction reflects a broader debate about how patterns of gene expression relate to evolutionary ancestry.^55^ A cell’s transcriptome can be conceptually divided into a small set of “regulatory” genes (including fate-related TFs) and a larger set of “effector” genes that carry out cellular functions. Both gene categories can inform evolutionary relationships,^21,56,57^ and for most vertebrate photoreceptors, they tell the same story (Figure S3). However, DC-A is unique in this respect: based on whole-transcriptome comparisons, DC-A is most similar to DC-P and red cones yet expresses multiple blue cone TFs (*SKOR1* and *FOXQ2*)^18^ (Figures 5D and 7B).

This pattern suggests that DC-A is a “molecular chimera,” unlike the “all-red” DC-P. Two evolutionary scenarios could explain this hybrid nature:

First, DC-A might have originated from a red cone duplication, preserving most effector genes while acquiring blue-cone-associated TFs through evolutionary drift. The plausibility of this scenario depends on how completely DC-A maintains the blue cone TF network, which merits further investigation.

Second, DC-A might have evolved from the ancestral blue cone while adapting to express red cone effector genes. Supporting this hypothesis, DC-A expresses blue-cone-enriched TFs *FOXQ2* and *SKOR1* and lacks the red-cone-enriched TFs *THRB* and *SAMD7*.^18^ Although we could not analyze *FOXQ2*—an essential gene for blue cone identity in fish^58^—due to its absence in standard chicken and lizard genomes, we confirmed the expression of other TFs reported by Liu et al.,^18^ including *SKOR1* (Figure 7B). DC-A also shares expression of the calcium-binding proteoglycan *SPOCK3* with blue cones. However, the regulatory picture is complex: *SKOR1* is not essential for blue cone development in zebrafish,^59^ and chicken DC-A expresses *RXRG*, which is necessary for the activation of LWS opsin in rodents.^60,61^ Resolving the precise transcriptional network governing DC-A development will require additional experimental work.

Several anatomical and functional properties provide additional context for these molecular findings. Supporting the red cone origin, DC-A uses the same light-detecting molecule (LWS opsin) that red cones use, and the overall gene expression pattern of DC-A is most similar to that of red cones and DC-P (Figures 6A, and S5). In birds, the synaptic outputs of DC-P and DC-A are physically organized together with those of rods in the outermost layer (layer 1) of the outer plexiform layer.^36,37^ They sit near red and green cones (in layer 2) but are separated from blue and UV cones (in layer 3).

Furthermore, both DC-P and DC-A form connections with a highly overlapping (though not identical) set of downstream neurons, often connecting alongside rods and red cones. This connection pattern follows “spectral block wiring,”^5,62^ the observation that postsynaptic neurons typically co-wire to “spectrally neighboring” photoreceptor types rather than skipping over intermediate ones. For example, if a neuron connects to both red and blue cone cells while ignoring the “spectrally intermediate” green cones, this would violate the principle—and such connections appear to be exceedingly rare (but see below).^36,37,63^ Another possibility consistent with a red cone origin is a stepwise diversification in which DC-A evolved from DC-P. Support for this hypothesis comes from the fact that DC-A’s closest transcriptional relative in both chicken and lizard is DC-P itself (Figures 6A, 6B, and S5). This similarity is driven by many genes in addition to *OPN1LW*, including *LRP2*, *HCN1*, *ISL2*, *PKM*, and *ARID3B* (Figure 7B).

Supporting a blue cone origin is the observation that DC-A shares patterns of cell-fate-determining TFs with blue cones that differ from all other cones, including red and DC-P (Figure S6).^18^ This relationship is further supported by experimental evidence showing that manipulation of the red-cone-fate-determining TF *THRB* during development eliminates red cones and DC-P but leaves DC-A intact.^18^ Moreover, recent connectomics studies identified a “candelabra-shaped” horizontal cell type in chicken and robin that largely bypasses red and green cones in layer 2 of the outer plexiform layer to connect primarily with DC-A in layer 1 and blue cones in layer 3.^17^ In addition, the typically elevated soma position of DC-A relative to DC-P mirrors the shifted soma positions observed in blue and red single cones in birds^36^ and teleost fish.^64,65^

From a functional perspective, the blue cone origin hypothesis gains additional support. Recent work suggests that among the four ancestral single cones of zebrafish, red and UV cones form the primary visual system, whereas green and blue cones have distinct roles: unlike red and UV, blue and green cones are not necessary or sufficient for “normal” vision and, instead, appear to represent the front end of a net-suppressive system that regulates the activity of red- and UV-cone-driven circuits.^5,66^ In this context, a coordinated duplication of red and blue single cones into DC-P and DC-A would create a new drive system (DC-P) with its own dedicated regulatory component (DC-A).^1^

### Fish “DCs”

One long-standing confusion concerns the presence of DCs in the retinas of teleost fish such as zebrafish.^14^ Here, our work bolsters previous assertions that the pairs of neurons referred to as DCs in fish and in tetrapods are evolutionarily distinct. “Fish DCs” are made up of pairs of ancestral red and green single cones, whereas “tetrapod DCs” represent an evolutionarily distinct set of cones that exist in parallel to ancestral red and green single cones. This distinction is elaborated in Baden et al.^15^

### Molecular relationships among photoreceptor types

The shared molecular signatures of the four ancestral single cones reinforce the widely accepted view that all cones share a common origin. Within zebrafish, chicken, and lizard we find that molecular similarities of cones consistently follow their spectral order, including when the analysis is restricted to TFs (Figures S7C–S7F): red → green → blue → UV. This finding was highly robust and could be recovered by an orthogonal analysis of DEG overlap (Figure 6B). This relationship hints that spectrally neighboring cones are evolutionarily related, which may provide a potential explanation for spectral block wiring (see above).^67,68^ Although we cannot rule out the alternative possibility that cones with similar spectral sensitivities converged on these gene expression patterns independently in the three species analyzed, we consider this scenario unlikely.

Our molecular analysis also shows that rods are equally dissimilar to all cone types (Figures 6A and S3), contrary to what previous studies of opsin protein sequences suggested.^2,41,42^ We found that rods express a distinct set of TFs and show no particular molecular relationship to green cones (Figures S6A and S6B). There are two possible explanations for this. First, rods may have evolved from a specific cone type but diverged so much that the molecular traces of this relationship are no longer detectable. Alternatively, rods may have evolved before cones underwent their spectral diversification (Figure 7A). The second hypothesis is supported by evidence from the pineal organ (a sister organ to the retina) in lower vertebrates, which possesses photoreceptors that resemble both rods and red cones but not the other cone types (e.g., Sapède and Cau^45^). Notably, recent analyses of the hagfish genome suggest that cyclostomes diverged from jawed vertebrates prior to the second whole-genome duplication.^3,69^ Given than lampreys have the rhodopsin ortholog, these findings suggest that rhodopsin is more ancient than previously thought.

Together, our results also bolster the long-standing notion that the ancestral type identity of vertebrate ciliary photoreceptors is stringently conserved over substantial phylogenetic distances that likely date back to the last common vertebrate ancestor, >500 mya.^5,70^ A parallel commentary summarizes this evidence to build the case for a new naming system of the vertebrate rods and cones.^15^ The new system will use the two-letter code “PR” (for photoreceptor), followed by a number: 0 for ancestral rods, 1–4 for the ancestral single cones in their spectral order starting from “red,” and 5 and 6 for the tetrapod DC (Figure 7A). The new nomenclature also provisions index 7 for the second/green rod of amphibians, whose transcriptomic identity and ancestral relationship remains unresolved.

## RESOURCE AVAILABILITY

### Lead contact

Further information and requests for resources and reagents should be directed to and will be fulfilled by the lead contact, Dr. Karthik Shekhar (kshekhar@berkeley.edu).

### Materials availability

This study did not generate new unique reagents.

### Data and code availability

- This paper analyzes existing, publicly available data, accessible at GEO: GSE239410, GSE175929, GSE159107, GSE237205, GSE237207, GSE237212, and GSE237204.
- Microscopy data reported in this paper will be shared by the lead contact upon request.
- All original code has been deposited on Zenodo at 10.5281/zenodo.15178568 and is also publicly available on Github at https://github.com/shekharlab/DoubleCones as of the date of publication.
- Any additional information required to reanalyze the data reported in this paper is available from the lead contact upon request.

## STAR★METHODS

### EXPERIMENTAL MODEL AND STUDY PARTICIPANT DETAILS

#### Animals

All procedures for *in situ* HCR were performed in accordance with the Washington University in St. Louis, Institutional Animal Care and Use Committee (IACUC) guidelines, and the UK Animals (Scientific Procedures) act 1986 and were approved by the animal welfare committee of the University of Sussex. We used female adult green anoles *Anolis carolinensis* (RRID: NCBITaxon_28377) purchased from Carolina Biological Supply, age unknown, and chickens age 3 to 5 days post hatching, sex unknown. Chickens *Gallus gallus* (RRID: NCBITaxon_9031) used for *in situ* were acquired from Joice and Hill Poultry Ltd.

### METHOD DETAILS

#### Selection of species

To investigate the evolution of DCs, we needed to sample 1) species that diverged before the emergence of DCs, 2) species with DCs, and 3) species that have since lost DCs:

For 1), we used zebrafish, which possesses the ancestral photoreceptor complement (rods plus red, green, blue, and UV cones). We attempted to include photoreceptors from goldfish (another teleost fish),^78^ but we were unable to identify blue and UV cones, possibly due to low sampling and/or poor opsin annotation.

For 2), we used chicken, lizard, and opossum, which contain DCs.^5^ We attempted to include the amphibian *Xenopus*,^79^ which also has DCs. Although we were able to identify several red cone clusters in *Xenopus*—indicating the likely presence of red cones and DCs—the overall cell count was too low for a comprehensive annotation.

For 3), we used two eutherian mammals: human^21^ and squirrel.^21^ We had first attempted to use mouse photoreceptors.^80,81^ However, some mouse cones are known to co-express both *OPN1MW* and *OPN1SW* along a dorsoventral gradient with *OPN1SW* enriched in the ventral retina.^50,51^ Due to this, we were unable to identify discrete populations of single-opsin cones (i.e. *OPN1MW*^+^ or *OPN1SW*^+^) across multiple mouse atlases,^80,81^ instead observing a single cluster with graded opsin expression. Therefore, we decided to use squirrel, in which we could easily distinguish the two cone clusters (Figure 2A).

#### Alignment of sc/snRNA-seq data

We retrieved pre-processed count matrices from published studies for zebrafish,^19,32^ chicken,^20^ brown anole lizard,^21^ opossum,^21^ squirrel,^21^ and human.^21^ However, we noticed that the lizard transcriptome did not contain annotations corresponding to the green and the blue opsin. Therefore, we re-aligned the raw sequencing data from lizard^21^ to a newer transcriptome assembly, *Anolis sagrei v2.1*, available on NCBI (https://www.ncbi.nlm.nih.gov/datasets/genome/GCF_025583915.1/). Table S1 lists the gene names corresponding to opsins in each species. As we could not identify DC components in opossum, we also attempted to re-align the opossum raw data to a newer NCBI assembly (mMonDom1.pri 2023) but this yielded similar results to the original ENSEMBL-aligned data.^21^ This suggests that more opossum cells are needed to resolve DC-P and DC-A.

#### Annotation of cell atlases

sc/snRNA-seq data clustering, integration and visualization was performed using Seurat v4.3.0^29^ in R v4.2.1.^82^ To ensure high-quality annotations, we applied a consistent pipeline to assemble the photoreceptor atlas for each species. This involved re-clustering the original dataset, filtering doublets, and annotating clusters based on the expression of opsin genes (Table S1). Clusters were annotated as rods, single cones (red, green, blue, UV) and putative DC-P and DC-A members. As noted in the main text, mammalian cone types were annotated based on their ancestry. Thus, the human red and green cones, which are both derived from ancestral red cones and express the LWS opsin,^5^ were annotated together as “red cones”; human blue cones, derived from the ancestral UV cone and express the SWS1 opsin, were annotated “UV cones”; and squirrel green cones, derived from ancestral red cones and express the LWS opsin, were annotated as “red cones” (Figures 2A and 2B).

For zebrafish, Ogawa and Corbo^32^ described an additional cone cluster defined by the co-expression of *opn1mw4* and *opn1lw1* opsin genes (*opn1mw4/opn1lw1*+ cones) in their scRNA-seq data. The authors hypothesized that this cluster might represent a unique subpopulation within the commonly observed R/G cone pairs in teleosts (see discussion). Ogawa and Corbo also noted the graded expression of *opn1lw1/2* and *opn1mw1/2/3/4* in the red and green cones, consistent with the presence of region-specific subpopulations. We were able to reproduce Ogawa and Corbo’s observations in the adult zebrafish snRNA-seq dataset of Lyu et al.^19^ (data not shown). However, we could not detect *opn1mw4/opn1lw1*^+^ cones in larval zebrafish at 4/5 days post fertilization, suggesting that *opn1mw4/opn1lw1*^+^ cones may arise at later developmental stages (data not shown). Finally, we note that the appearance of *opn1mw4/opn1lw1*^+^ cones as a single cluster in both scRNA-seq and snRNA-seq datasets suggests that they represent a subpopulation of single cones rather than true double cones.

#### Distinguishing intact DCs from DC-P and DC-A

In their scRNA-seq atlas of the chicken retina, Yamagata et al.^20^ identified three putative double cone clusters - $DC_{a},$ $DC_{b}$, and $DC_{c}$. We hypothesized that one of these may represent intact DCs, with the other two being dissociated DC-P and DC-A cells. To test this, we used linear regression to model the gene expression of each cluster as a linear combination of the other two clusters (e.g., $DC_{b}\approx \alpha DC_{a}+\beta DC_{c}+\gamma$), where $DC_{i}$ is the gene expression vector (i=a,b,c),α and β are regression coefficients, and γ is the bias. We found that $DC_{b}\approx 0.5DC_{a}+0.56DC_{c}$ (*p* < 10^−3^, Figure S1D), suggesting that $DC_{b}$ represents the intact double cone and $DC_{a}$ and $DC_{c}$ are its members. Consistent with this result, modeling either $DC_{a}$ or $DC_{c}$ as a linear combination of the other two clusters yielded subtractive combinations (Figure S1D), again consistent with $DC_{b}$ representing full double cones. Thus, we re-annotated $DC_{b}$ as full intact DCs and omitted it from downstream analysis. Notably, clusters corresponding to intact DCs were found only in scRNA-seq data, not snRNA-seq data. We used chicken as the basis for annotation of the less-studied anole lizard. We hypothesized that the DC cluster expressing the glycogen-related gene *STBD1* represents DC-A because DC-A has an enlarged glycogen-containing paraboloid, leaving the *THRB*^+^*STBD1*^−^*MYLK*^−^ cluster as the DC-P. We later confirmed these annotations using histology (Figures 5A–5D and S4A–S4H).

#### Cross-species photoreceptor alignment using SAMap

SAMap v1.0.15^30^ was run in Python v3.9.19. SAMap analysis was run as follows. 1) An initial SAMap object was instantiated and the resulting BLAST homology graph was saved for later use, as this is the slowest step. 2) h5ad count files exported from Seurat were preprocessed using SAM^83^ with 100 PCs, k=20 nearest neighbors, and 3000 genes. 3) SAMap was run pairwise for 3 iterations, with computing neighborhoods from keys set to true. 4) Alignment scores (average scores from the kNN graph) were extracted using the get_mapping_scores function in SAMap. We also used the function GenePairFinder with default parameters to identify conserved gene markers for photoreceptors in Figures 3D and 5C.

Due to the large differences in photoreceptor type frequency across species, we downsampled each photoreceptor type within each species to 100 cells. This inherently introduces randomness in the mapping, so we repeated SAMap experiments 50 times with different random samples and took the mean alignment scores across the 50 runs. This ensured that our results were robust and reproducible across different random samples of cells. To estimate the 0.05 and 0.10 significance level in Figure 5D, we used the putatively erroneous, cross-type alignment scores from the five-species integration of ancestral photoreceptors in Figure 3C (e.g. red cone vs green cone, blue cone vs rod, etc.).

#### Transcription factor gene sets

For analysis SAMap and PCA analyses restricted to transcription factors (TFs), we used gene sets from two sources: 1) 885 TFs from AnimalTFDB 3.0 for *Gallus gallus*,^39^ and 2) 112 transcription-related genes that were differentially expressed across photoreceptor types in Liu et al.^18^ The AnimalTFDB set was intended to be more permissive and thus used homologs, while the Liu et al. set was intended to be more stringent and used 1:1 orthologs. For both sets, we used a reciprocal BLAST approach to retrieve either homologs (AnimalTFDB) or orthologs (Liu et al.) in each species studied here. Briefly, we BLASTed the chicken protein sequences to the other species. For AnimalTFDB, we BLASTed the hits (bit-score > 100, e-value < 1 × 10^−25^) back to chicken to expand the TF space in chicken. The number of TFs used for the AnimalTFDB set were: 2090 (zebrafish); 1254 (chicken); 1886 (lizard); 1298 (squirrel); 1595 (human). These numbers vary widely due to the number of protein sequences available for these genome assemblies. The number of TFs used for the Liu et al. set were: 94 (zebrafish); 106 (chicken); 99 (lizard); 89 (squirrel); 99 (human).

#### Hierarchical clustering and principal component analysis (PCA)

Hierarchical clustering trees based on DEGs (Figures S5K–S5P) were constructed using the number of DEGs as the distance. The transcription factor pattern analysis in Figure S6 was performed similarly to Liu et al.,^18^ with the main difference being that we used the percentage of cells expressing (PE) the gene instead of average expression. The seven photoreceptor types were ordered from highest to lowest PE value, and the PE differences along this ranked list were computed. TFs were classified as enriched in one or more types if its expression was > 10% higher than in the next type in the ordered series. Although *ISL2* was slightly below this cutoff in lizard, we still included it for lizard since it showed a similar pattern to that in chicken.

For the PCAs within species (Figure 6A), we used the pseudobulked expression of the top 2000 highly variable genes to compute principal components (PCs). Normally, PCA is preceded by scaling across features. We observed that when computing PCs with centered and scaled expression data, the resulting PCs were dominated by contributions from noisy, lowly expressed genes. We found two strategies to avoid this noise. 1) We centered the data but did not scale prior to PCA. This effectively gives highly expressed genes more importance in the reduced dimension. 2) We require genes to be expressed above a certain threshold in one cluster (e.g. 5–15% of cells). These two methods led to very similar results, so we used centering without scaling since it did not require arbitrary hyperparameters, and was faster. The overall qualitative features of the PCA embedding remained robust when excluding opsin genes and restricting the analysis to TFs (Figures S7C–S7F).

For the joint PCA in Figure 6A, we used the homology graph from SAMap^30^ to transform the gene expression values of zebrafish and lizard into the chicken gene expression space. Briefly, the homology graph A is an $m_{1}\times m_{2}$ matrix, where $m_{1}$ and $m_{2}$ are the number of genes from species 1 and species 2 respectively, and whose entries denote the similarity between genes across species. These similarities are initialized from protein BLAST similarity, but then refined based on expression similarity.^30^ If $X\in R^{m_{1}\times n_{1}}$ is the gene-by-type expression matrix for species 1 with $n_{1}$ types, then $A^{T}X\in R^{m_{2}\times n_{1}}$ is the transformed gene-by-type matrix in the expression space of species 2. We scaled within each species to remove extensive batch effects across species, then subsetted to genes that are present across all three species (~8000 genes), and horizontally concatenated the three gene-by-type matrices. To mitigate species-specific noise, we binarized these scaled expression values by setting genes whose expression was above an arbitrary threshold to 1 and the rest to 0. We used a threshold of −0.15, but other values around 0 gave similar results. We then ran PCA on the resulting binarized gene-by-type matrix.

#### Immunohistochemistry

Chicken tissue for immunohistochemistry was a gift from Christine Wildsoet. Eyes from a 7-day post-hatch chicken were enucleated immediately post-mortem as previously described.^71^ The anterior segments were removed, and the retina/choroid complex was fixed in 4% paraformaldehyde for 30 minutes at 22°C. Samples were cryoprotected in graded sucrose solutions (10%, 20%, 30%) and stored at −20°C until use. For immunostaining, retinas were washed in phosphate-buffered saline (PBS) for 15 minutes, then blocked for 1 hour in 10% normal horse serum (NHS), 1% Triton X-100, and 0.025% NaN_3_ in PBS at 22°C. Primary antibodies, mouse anti-Calbindin D-28K (Synaptic Systems CB300, 1:250, RRID: AB_3542811) and rabbit anti-MYLK (Invitrogen PA5–79716, 1:300, RRID: AB_2746831), were diluted in 3% NHS, 1% Triton X-100, 0.025% NaN_3_ and applied for 3 days at 22°C. The samples were washed with PBS, then incubated overnight with secondary antibodies, Donkey anti-rabbit Alexa Fluor 488 (Molecular Probes A21206, 1:800, RRID: AB_2535792) and Donkey anti-mouse Alexa Fluor 594 (Molecular Probes A-21203, 1:800, RRID: AB_141633), diluted in 3% NHS, 0.1% Triton X-100, 0.025% NaN_3_. After final washes, residual choroid was removed, and the sample was mounted photoreceptor side up in Mowiol (Sigma) mounting medium.

For imaging CALB1 and MYLK in DCs, confocal laser scanning images were acquired on a Zeiss LSM 880 microscope with a Zeiss Plan-Apochromat 63×/1.4 oil DIC objective. Images were acquired at 7.6 pixels per μm resolution. Z-stacks were taken with a step size of 1 μm to identify the inner segments, which were imaged approximately 2–3 μm below the oil droplet that delimits the inner and outer segments. We used an excitation wavelength of 488 nm for Alexa-488 (MYLK) and 594 nm for Alexa-594 (CALB1). Adjustments to brightness, contrast and pseudo-color were made in FIJI.

#### *In situ* Hybridization Chain Reaction (HCR)

Adult lizards were humanely euthanized with a high dose of anesthesia (Alfaxalone 60 mg/kg) subcutaneous injection and chickens were humanely sacrificed by cervical dislocation followed cutting of the aorta. For both species, retinal tissues were dissected from the enucleated whole eyes by removing cornea, lens and epithelial layer in 1x PBS. The tissues were immediately fixed in 4% paraformaldehyde (Agar Scientific, AGR1026) in PBS for 60 min at room temperature, followed by three washes in PBS. The tissues were then sliced at 200 μm thickness using a tissue chopper. The standard *in situ* HCR was performed according to the manufacturer’s protocol using HCR Probe hybridization buffer, Probe Wash buffer, and Amplification buffer (Molecular Instruments). HCR probe sets and Amplifiers were custom-designed (Table S2). Hoechst 33342 was added during the wash step after the amplification step to visualize nuclei. Confocal image stacks were taken immediately after the *in situ* HCR on a FV1000 microscope (Olympus) with a 40x oil immersion objective (HC PL APO CS2, Leica). Typical voxel size was 0.62 μm and 0.5 μm in the *x* -*y* and z, respectively. Contrast, brightness and pseudo-color were adjusted for display in FIJI.^74^

#### Data visualization

Animal drawings used throughout the paper can be found at https://github.com/BadenLab/Free_to_use_vector_graphics. Plots were generated using the R package ggplot2 v3.5.1.^72^ Heatmaps were generated using the R package ComplexHeatmap v2.20.0.^73^ Sankey diagrams were generated using the R package ggforce v0.4.2.^75^ Upset plots in Figure S6B were generated using the R package UpSetR v1.4.0.^76^

### QUANTIFICATION AND STATISTICAL ANALYSIS

#### Differential gene expression analysis

For identifying differentially expressed genes (DEGs), we used the R package presto. For constructing the hierarchical clustering trees in Figures S5K–S5P, we used pairwise DEGs with an average log_2_ fold change cutoff of 1 and Benjamini-Hochberg-adjusted p-value cutoff of 0.001. For exploration of DEGs distinguishing DC members from each other and from red and blue cones (Figures S5A–S5J; Data S1), we used more permissive cutoffs (average log_2_ fold change cutoff of 0.25 and Benjamini-Hochberg-adjusted p-value cutoff of 0.01).

#### Quantification of positive cells in situ HCR

Puncta were detected by thresholding the image stacks followed by 3D Object Counter in FIJI. For *OPN1LW* in anole lizards, nuclei with puncta signal were counted as positive. Briefly, we used Cellpose^77^ to segment the nuclei, and then used a script from the 10x Genomics Xenium pipeline (https://www.10xgenomics.com/analysis-guides/performing-3d-nucleus-segmentation-with-cellpose-and-generating-a-feature-cell-matrix) to assign each puncta to a nuclei. For *STBD1* in anole lizards, the density of puncta was too low to restrict analysis to solely the nuclei. Instead, the density of puncta at each voxel was computed to create a density map for *STBD1*, and high-density regions were counted as *STBD1*^+^ cells.

## Supplementary Material

Supplementary figures **Figure S1: Identification of principal, accessory, and full double cones in the chicken retinal atlas, related to** Figure 2. A) 2-D UMAP embedding of chicken retinal atlas^S1^. Three double cone (DC) clusters annotated by Yamagata et al., labeled DCa, DCb, and DCc, are highlighted. B) Violin plot showing the normalized and log-transformed expression values for marker genes in the photoreceptor clusters in panel A. Shown are the rod marker *RHO*, the cone marker *PDE6H*, the ancestral cone marker *ZEB2*, the double cone marker *CALB1*, the red cone marker *THRB*, and a novel marker for DCa (*STBD1*). Notice that DCb expresses *THRB* and *STBD1* at intermediate levels compared to DCa and DCc. C) Pairwise gene expression correlations between DCa, DCb, and DCc. DCb is similar to both DCa and DCc. Gene expression correlation of DCa, DCb, and DCc to the best-fit linear combination of the other two clusters. Middle panel shows that DCb is an average of DCa and DCc, indicating that it represents full, intact double cones (both principal and accessory member) entering a single 10x droplet. E) Relative proportions of photoreceptor types from scRNA-seq of E18 chicken retina^S1^. In these calculations, we estimated the total number of double cones to be the sum of the number of intact double cones (DCb) and the average of the numbers of the principal (DCc) and accessory (DCa) cells. F) Relative proportions of photoreceptor types from immunostaining of P15 chicken (Figure 2B of Kram et al. 2010^S2^, reproduced using automeris.io WebPlotDigitizer). Photoreceptor types are colored the same as in panel **E**. The x-axis shows the proportions for tissue sections obtained from different quadrants of the retina: dorsonasal (DN), dorsotemporal (DT), ventronasal (VN), ventrotemporal (VT). Differences in proportions between panels C and D may be due to biases in cell capture in scRNA-seq and/or due to differences in age (E18 vs. P15).
**Figure S2: Conserved transcriptional signatures for rods and single cones, related to** Figure 3. Heatmap of scaled gene expression showing the top conserved transcriptional signatures of ancestral photoreceptors (same as Figure 3D, but transposed, and gene names listed). Gene names are in the same order as the species (zebrafish, chicken, lizard, squirrel, and human). SAMap identified known paralogous relationships; for example, *opn1mw1/2/3* in zebrafish. Missing genes are indicated as *NA*.
**Figure S3: Heatmaps of SAMap alignment scores from integration experiments, related to** Figures 3 and 5. A-D) Heatmaps of SAMap alignment scores for the integration of single cones and rods in the five-species integration. E-H) Heatmaps of SAMap alignment scores for the zebrafish, chicken, lizard integration including DCs. Values shown are means from 50 experimental runs with different subsamples of cells. Experiments were run using four different gene sets. A and E) all genes. B and F) all genes minus visual opsins. C and G) ~1500 TFs from AnimalTFDB 3.0^S3^. D and H) ~100 highly variable TFs from Liu et al.^S4^.
**Figure S4: Analysis of photoreceptor proportions in chicken and green anole lizard (*Anolis carolinensis*), related to** Figure 4. A) *In situ* Hybridization Chain Reaction (HCR) targeting *STBD1* (magenta) and *CALB1* (cyan) in retinal cross sections of chicken (3–5 days post-hatch). Nuclei are stained by Hoechst (grey). Arrows indicate photoreceptors positive for *STBD1* and/or *CALB1*. OS, outer segment; IS, inner segment; NL, nuclear layer. B) Same as A, but targeting *OPN1LW* (red) and nuclei (grey) in retinal cross sections of chicken. C) Percentage of *OPN1LW*^+^ photoreceptors in the NL. Each circle is a biological replicate. A total of 409 nuclei were counted in the NL and the typical field of view was 320×100 μm. D) Same as C, but the percentage of *STBD1*^+^ photoreceptors in the ONL. A total of 423 nuclei were counted in the NL. Although HCR tends to underestimate the absolute proportions of *OPN1LW*^+^ cells compared to the chicken atlas (HCR: ~50%; atlas: ~66%), the ratio of *OPN1LW*^+^ to *STBD1*^+^ cells is consistent (HCR: 2.5; atlas: 2.2). E) *In situ* HCR targeting *OPN1LW* in retinal cross sections of the green anole lizard (left panel). RNA signal and nuclei (Hoechst) are represented in magenta and grey, respectively. In the inset (middle), *OPN1LW*^+^ nuclei are highlighted in cyan (details in STAR METHODS). Right panel shows *OPN1LW*^+^ nuclei in the same field of view as the left panel. ONL, outer nuclear layer; INL, inner nuclear layer; GCL, ganglion cell layer. F) Percentage of *OPN1LW*^+^ nuclei in the ONL. Each circle is a biological replicate. A total of 646 nuclei were counted in the ONL and the typical field of view was 640×540 μm. G) Same as A, but for HCR experiments targeting *STBD1*. Regions of high HCR signal density are shown in cyan. H) Percentage of *STBD1*^+^ nuclei in the ONL. Each circle is a biological replicate. A total of 407 nuclei were counted in the ONL and the typical field of view was 640×380 μm. Although HCR tends to underestimate the absolute proportions of *OPN1LW*^+^ cells compared to the lizard atlas (HCR: ~70%; atlas: ~90%), the ratio of *OPN1LW*^+^ to *STBD1*^+^ cells is consistent (HCR: 5.8; atlas: 5.6).
**Figure S5: Genes that distinguish DC-P from DC-A, and DC-P or DC-A from red and blue cones, related to** Figure 5. Scatterplots comparing normalized and log-transformed gene counts between two selected PR clusters in chicken (A-E) and lizard (F-J). Chicken: A) DC-P vs DC-A. B) DC-P vs red. C) DC-A vs red. D) DC-P vs blue. E) DC-A vs blue. Lizard: F) DC-P vs DC-A. G) DC-P vs red. H) DC-A vs red. I) DC-P vs blue. J) DC-A vs blue. Top 30 differentially expressed genes (DEGs) are labeled – a full list is provided in Table S2. B) Heatmaps showing number of pairwise differentially expressed genes (Benjamini-Hochberg-adjusted p < 0.001 and log_2_ fold change change > 1) between clusters for each species. Hierarchical clustering was performed using complete linkage and the number of DEGs as the distance.
**Figure S6: Analysis of transcription factor (TF) patterns in chicken and lizard photoreceptor types, related to** Figure 5. A) Dot plot showing scaled expression values as the color and percentage of cells with expression as the size. *Left panel:* Chicken TFs expressed in at least 15% of cells in at least one cluster. TFs are sorted by expression pattern. *Right panel:* Lizard TFs expressed in at least 15% of cells in one cluster. TFs are sorted by pattern. The hierarchical clustering tree for the genes was constructed from the binarized expression matrix using Manhattan distances and complete linkage. B) Upset plots^S5^ showing the size of the various TF patterns observed in **A** Chicken TFs (top) and Lizard TFs (bottom).
**Figure S7: Robustness of principal component 2 in various datasets and conditions, related to** Figure 6. A) PCA of averaged photoreceptor gene expression profiles within three zebrafish datasets (columns)^S6,S7^. In all three cases, *PC*1 separates rods and cones, while *PC*2 separates cone types based on their color. B) Scatterplots comparing *PC*2 gene contributions (loadings) between pairs of datasets in panel **A**. C-F) Principal component analysis (PCA) of zebrafish, chicken, and lizard photoreceptor gene expression with different gene sets: C) PCA with all genes (exactly like Figure 6A). D) PCA with all genes minus visual opsins. E) Using only ~1500 TFs from AnimalTFDB 3.0. F) Using only ~100 highly variable TFs from Liu et al.^S4^. G) Dot plot showing the top 50 positive and top 50 negative gene contributors to PC2 from chicken in panel **C**. Color shows average scaled expression while dot size shows the percentage of cells with expression. Value shown in parentheses next to gene shows the PC loading value (contribution to the PC).

Supp Table 1

Supp Table 2

Supp Table 3

SUPPLEMENTAL INFORMATION

Supplemental information can be found online at https://doi.org/10.1016/j.cub.2025.03.060.

## Figures and Tables

**KEY RESOURCES TABLE:** 

REAGENT or RESOURCE	SOURCE	IDENTIFIER

Antibodies		

Mouse anti-Calbindin D-28K	Synaptic Systems	CAT# CB300; RRID: AB_3542811
Rabbit anti-MYLK	Invitrogen	CAT# PA5-79716; RRID: AB_2746831
Donkey anti-rabbit Alexa Fluor 488	Molecular Probes	CAT# A21206; RRID: AB_2535792
Donkey anti-mouse Alexa Fluor 594	Molecular Probes	CAT# A-21203; RRID:AB_141633

Biological samples		

Chicken tissue for immunohistochemistry	Christine Wildsoet; Zhang et al.^71^	RRID: NCBITaxon_9031
Chicken tissue for in situ hybridization chain reaction	Joice and Hill Poultry Ltd.	https://www.joiceandhill.co.uk/en/
Green anole lizard tissue for in situ hybridization chain reaction	Carolina Biological	RRID: NCBITaxon_28377

Deposited data		

Processed data files for snRNA-seq of zebrafish retinal cells	Lyu etal.^19^	GEO: GSE239410
Processed data files for scRNA-seq of zebrafish retinal cells	Ogawa and Corbo^32^	GEO: GSE175929
Processed data files for scRNA-seq of chicken retinal cells	Yamagata et al.^20^	GEO: GSE159107
Processed data files for snRNA-seq of lizard retinal cells	Hahn et al.^21^	GEO: GSE237205
Processed data files for snRNA-seq of opossum retinal cells	Hahn et al.^21^	GEO: GSE237207
Processed data files for snRNA-seq of squirrel retinal cells	Hahn et al.^21^	GEO: GSE237212
Processed data files for snRNA-seq of human retinal cells	Hahn et al.^21^	GEO: GSE237204

Oligonucleotides		

See Table S2 for in situ HCR probes	Molecular Instruments	N/A

Software and algorithms		

Seurat	Stuart et al.^29^	https://satijalab.org/seurat/
SAMap	Tarashansky et al.^30^	https://github.com/atarashansky/SAMap
ggplot2	Wickham^72^	https://ggplot2.tidyverse.org/
ComplexHeatmap	Gu et al.^73^	https://github.com/jokergoo/ComplexHeatmap?tab=readme-ov-file
FIJI	Schindelin et al.^74^	https://imagej.net/software/fiji/
Ggforce	Pedersen^75^	https://ggforce.data-imaginist.com
UpSetR	Conway et al.^76^	https://github.com/hms-dbmi/UpSetR
Cellpose	Stringer et al.^77^	https://www.cellpose.org

## Highlights

- Single-cell transcriptomic analysis of photoreceptor orthology among vertebrates
- Molecular classification of the double-cone principal and accessory members
- The principal member is transcriptomically similar to ancestral red cones
- Cone transcriptomic variation mirrors spectral relationships
