# Supplementary material for: Comparative transcriptomic insights into the evolution of vertebrate photoreceptor types
Source: Curr Biol. Author manuscript; Available in PMC 2026 Jul 4. (PMC13332834; doi:10.1016/j.cub.2025.03.060)
Supplement: Supplementary figures — Figure S1: Identification of principal, accessory, and full double cones in the chicken retinal atlas, related to Figure 2. A) 2-D UMAP embedding of chicken retinal atlasS1. Three double cone (DC) clusters annotated by Yamagata et al., labeled DCa, DCb, and DCc, are highlighted. B) Violin plot showing the normalized and log-transformed expression values for marker genes in the photoreceptor clusters in panel A. Shown are the rod marker RHO, the cone marker PDE6H, the ancestral cone marker ZEB2, the double cone marker CALB1, the red cone marker THRB, and a novel marker for DCa (STBD1). Notice that DCb expresses THRB and STBD1 at intermediate levels compared to DCa and DCc. C) Pairwise gene expression correlations between DCa, DCb, and DCc. DCb is similar to both DCa and DCc. Gene expression correlation of DCa, DCb, and DCc to the best-fit linear combination of the other two clusters. Middle panel shows that DCb is an average of DCa and DCc, indicating that it represents full, intact double cones (both principal and accessory member) entering a single 10x droplet. E) Relative proportions of photoreceptor types from scRNA-seq of E18 chicken retinaS1. In these calculations, we estimated the total number of double cones to be the sum of the number of intact double cones (DCb) and the average of the numbers of the principal (DCc) and accessory (DCa) cells. F) Relative proportions of photoreceptor types from immunostaining of P15 chicken (Figure 2B of Kram et al. 2010S2, reproduced using automeris.io WebPlotDigitizer). Photoreceptor types are colored the same as in panel E. The x-axis shows the proportions for tissue sections obtained from different quadrants of the retina: dorsonasal (DN), dorsotemporal (DT), ventronasal (VN), ventrotemporal (VT). Differences in proportions between panels C and D may be due to biases in cell capture in scRNA-seq and/or due to differences in age (E18 vs. P15). Figure S2: Conserved transcriptional signatures for rods and single cones, relat [file NIHMS2182580-supplement-Supplementary_figures.pdf]

Current Biology, Volume *35*

Supplemental Information

Comparative transcriptomic insights into the evolution of vertebrate photoreceptor types

Dario Tommasini, Takeshi Yoshimatsu, Teresa Puthussery, Tom Baden, and Karthik Shekhar

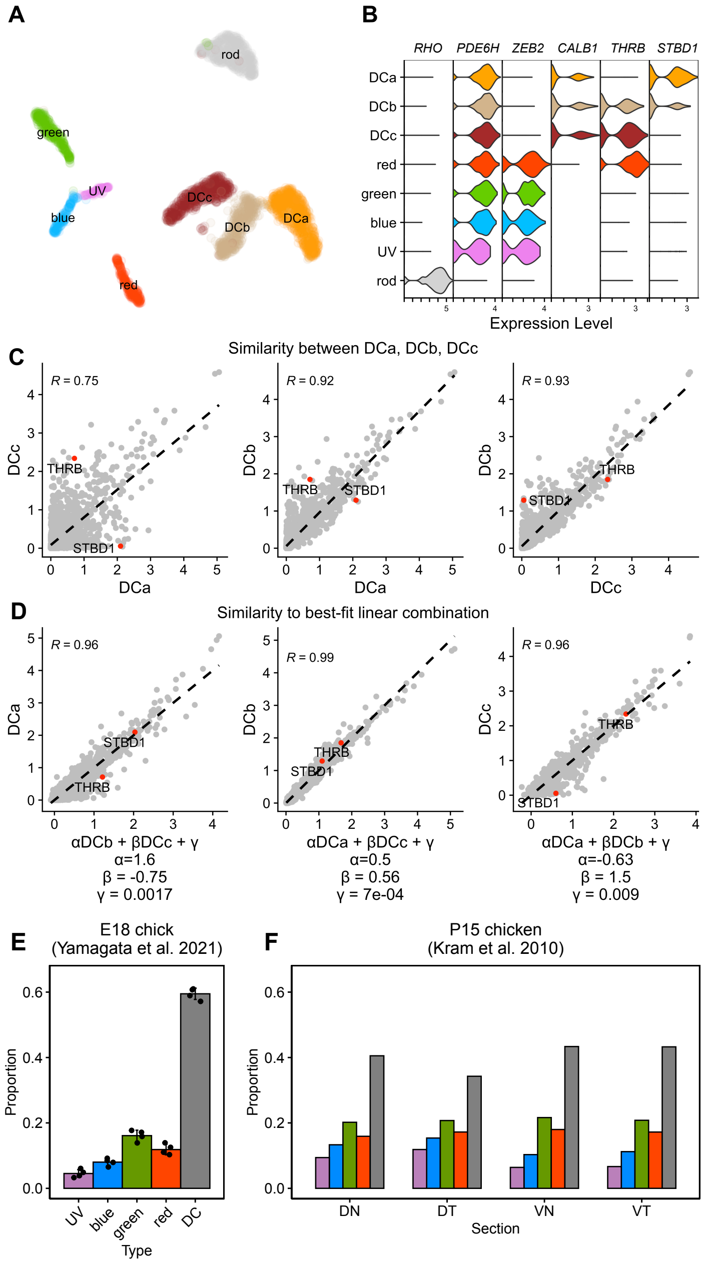

Figure S1: Identification of principal, accessory, and full double cones in the chicken retinal atlas, related to Figure 2. A) 2-D UMAP embedding of chicken retinal atlas[S1]. Three double cone (DC) clusters annotated by Yamagata et al., labeled DCa, DCb, and DCc, are highlighted. B) Violin plot showing the normalized and log-transformed expression values for marker genes in the photoreceptor clusters in panel A. Shown are the rod marker *RHO*, the cone marker *PDE6H*, the ancestral cone marker *ZEB2*, the double cone marker *CALB1*, the red cone marker *THRB*, and a novel marker for DCa (*STBD1*). Notice that DCb expresses *THRB* and *STBD1* at intermediate levels compared to DCa and DCc. C) Pairwise gene expression correlations between DCa, DCb, and DCc. DCb is similar to both DCa and DCc. Gene expression correlation of DCa, DCb, and DCc to the best-fit linear combination of the other two clusters. Middle panel shows that DCb is an average of DCa and DCc, indicating that it represents full, intact double cones (both principal and accessory member) entering a single 10x droplet. E) Relative proportions of photoreceptor types from scRNA-seq of E18 chicken retina[S1]. In these calculations, we estimated the total number of double cones to be the sum of the number of intact double cones (DCb) and the average of the numbers of the principal (DCc) and accessory (DCa) cells. F) Relative proportions of photoreceptor types from immunostaining of P15 chicken (Figure 2B of Kram et al. 2010[S2], reproduced using automeris.io WebPlotDigitizer). Photoreceptor types are colored the same as in panel **E**. The x-axis shows the proportions for tissue sections obtained from different quadrants of the retina: dorsonasal (DN), dorsotemporal (DT), ventronasal (VN), ventrotemporal (VT). Differences in proportions between panels C and D may be due to biases in cell capture in scRNA-seq and/or due to differences in age (E18 vs. P15).

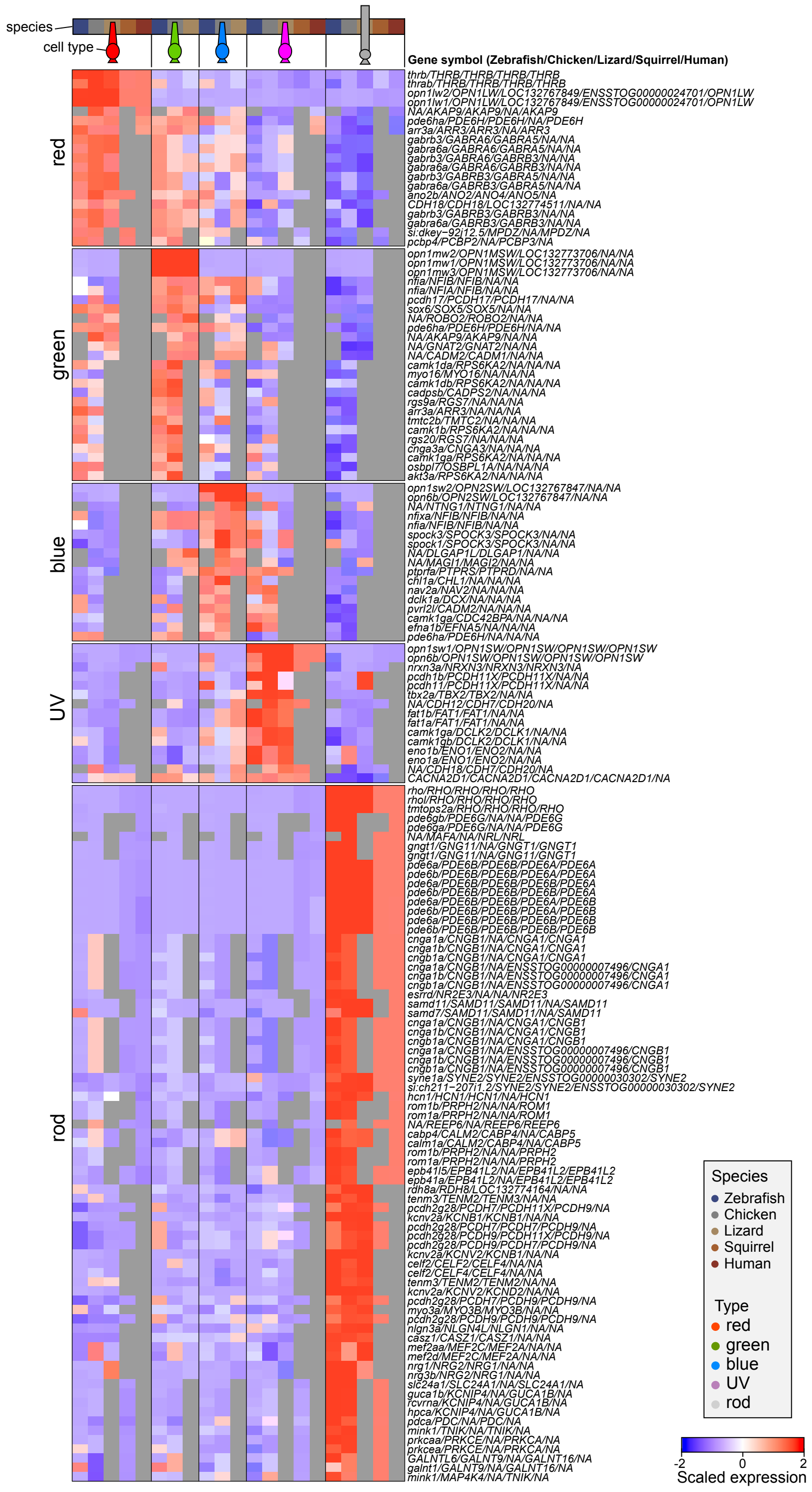

Figure S2: Conserved transcriptional signatures for rods and single cones, related to Figure 3. Heatmap of scaled gene expression showing the top conserved transcriptional signatures of ancestral photoreceptors (same as **Figure 3D**, but transposed, and gene names listed). Gene names are in the same order as the species (zebrafish, chicken, lizard, squirrel, and human). SAMap identified known paralogous relationships; for example, *opn1mw1/2/3* in zebrafish. Missing genes are indicated as *NA*.

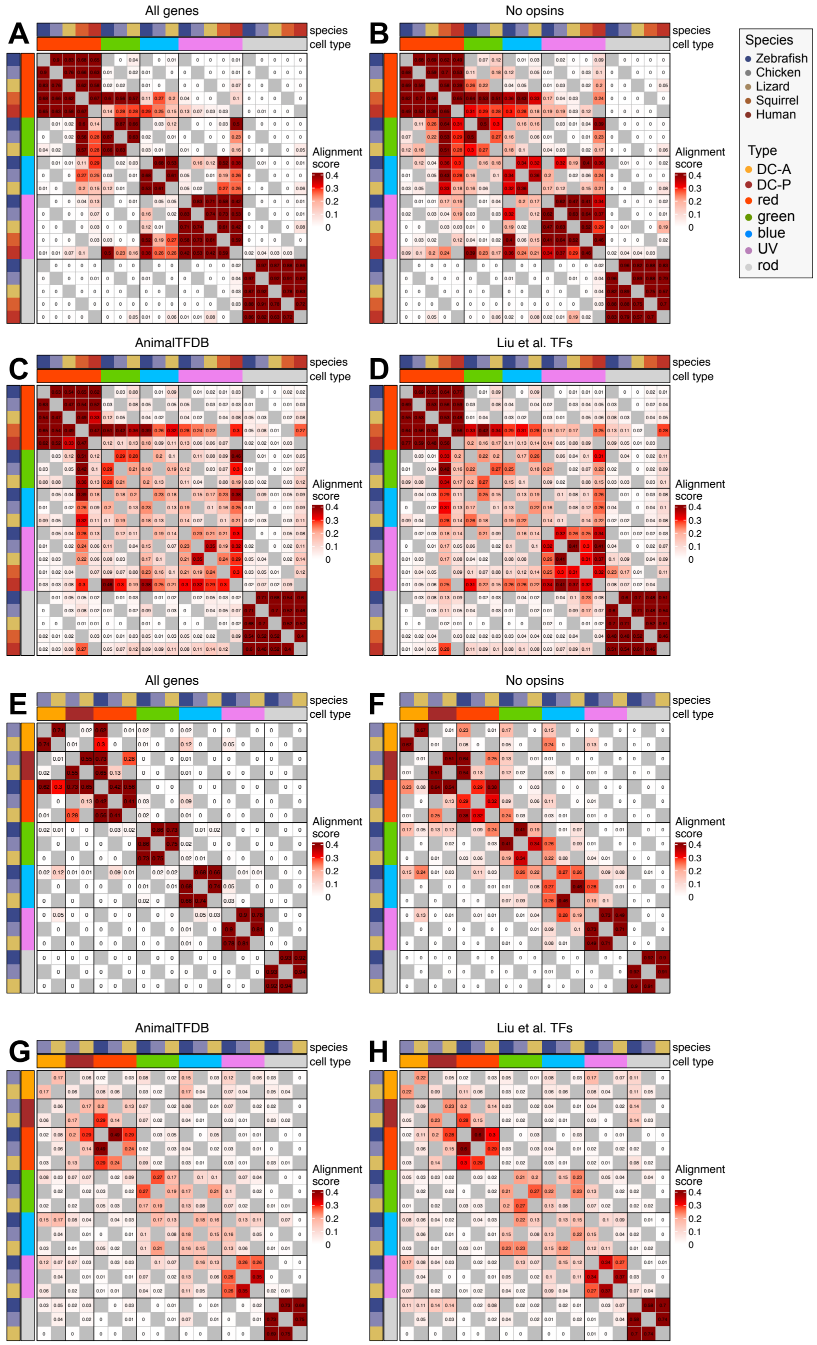

Figure S3: Heatmaps of SAMap alignment scores from integration experiments, related to Figures 3 and 5. A-D) Heatmaps of SAMap alignment scores for the integration of single cones and rods in the five-species integration. E-H) Heatmaps of SAMap alignment scores for the zebrafish, chicken, lizard integration including DCs. Values shown are means from 50 experimental runs with different subsamples of cells. Experiments were run using four different gene sets. A and E) all genes. B and F) all genes minus visual opsins. C and G) ∼1500 TFs from AnimalTFDB 3.0[S3]. D and H) ∼100 highly variable TFs from Liu et al.[S4].

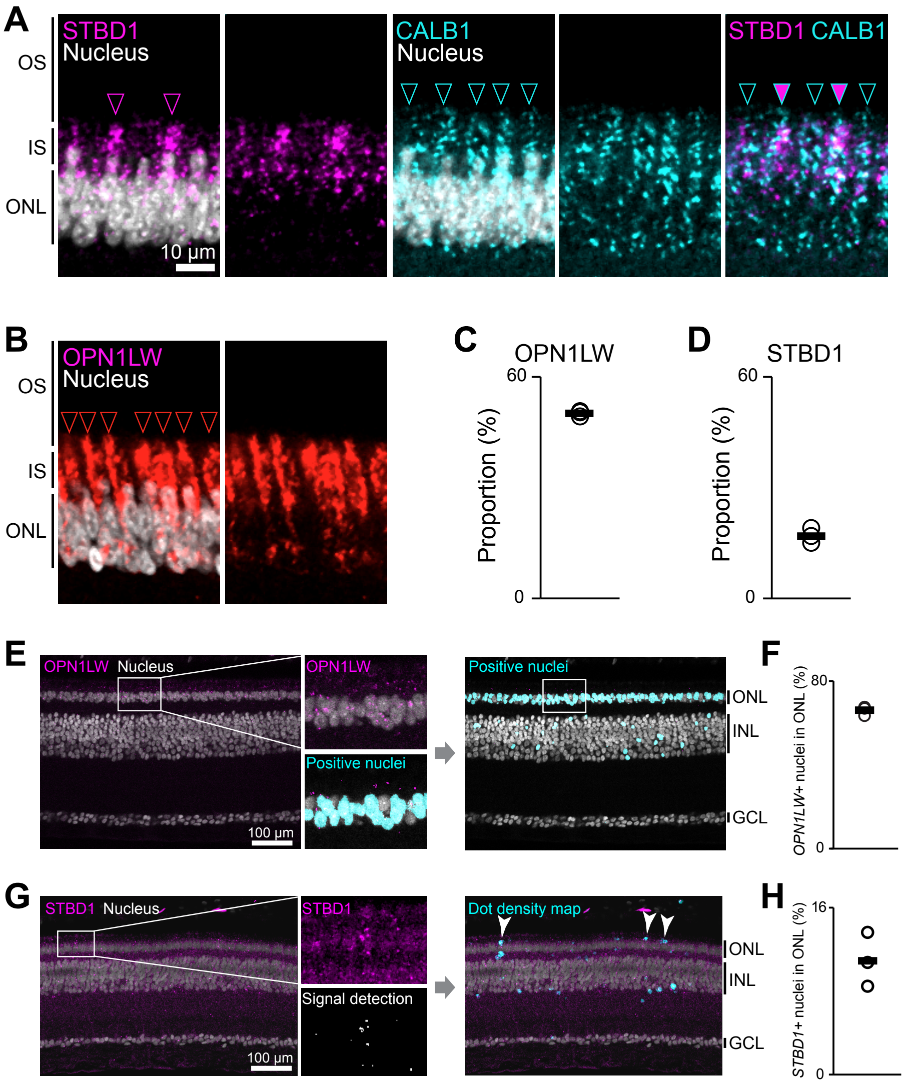

Figure S4: Analysis of photoreceptor proportions in chicken and green anole lizard (*Anolis carolinensis*), related to Figure 4. A) *In situ* Hybridization Chain Reaction (HCR) targeting *STBD1* (magenta) and *CALB1* (cyan) in retinal cross sections of chicken (3-5 days post-hatch). Nuclei are stained by Hoechst (grey). Arrows indicate photoreceptors positive for *STBD1* and/or *CALB1*. OS, outer segment; IS, inner segment; NL, nuclear layer. B) Same as A, but targeting *OPN1LW* (red) and nuclei (grey) in retinal cross sections of chicken. C) Percentage of *OPN1LW*$^+$ photoreceptors in the NL. Each circle is a biological replicate. A total of 409 nuclei were counted in the NL and the typical field of view was 320x100 μm. D) Same as C, but the percentage of *STBD1*$^+$ photoreceptors in the ONL. A total of 423 nuclei were counted in the NL. Although HCR tends to underestimate the absolute proportions of *OPN1LW*$^+$ cells compared to the chicken atlas (HCR: $\sim$50% ; atlas: $\sim$66%), the ratio of *OPN1LW*$^+$ to *STBD1*$^+$ cells is consistent (HCR: 2.5; atlas: 2.2). E) *In situ* HCR targeting *OPN1LW* in retinal cross sections of the green anole lizard (left panel). RNA signal and nuclei (Hoechst) are represented in magenta and grey, respectively. In the inset (middle), *OPN1LW*$^+$ nuclei are highlighted in cyan (details in **STAR METHODS**). Right panel shows *OPN1LW*$^+$ nuclei in the same field of view as the left panel. ONL, outer nuclear layer; INL, inner nuclear layer; GCL, ganglion cell layer. F) Percentage of *OPN1LW*$^+$ nuclei in the ONL. Each circle is a biological replicate. A total of 646 nuclei were counted in the ONL and the typical field of view was 640x540 μm. G) Same as A, but for HCR experiments targeting *STBD1*. Regions of high HCR signal density are shown in cyan. H) Percentage of *STBD1*$^+$ nuclei in the ONL. Each circle is a biological replicate. A total of 407 nuclei were counted in the ONL and the typical field of view was 640x380 μm. Although HCR tends to underestimate the absolute proportions of *OPN1LW*$^+$ cells compared to the lizard atlas (HCR: $\sim$70%; atlas: $\sim$90%), the ratio of *OPN1LW*$^+$ to *STBD1*$^+$ cells is consistent (HCR: 5.8; atlas: 5.6).

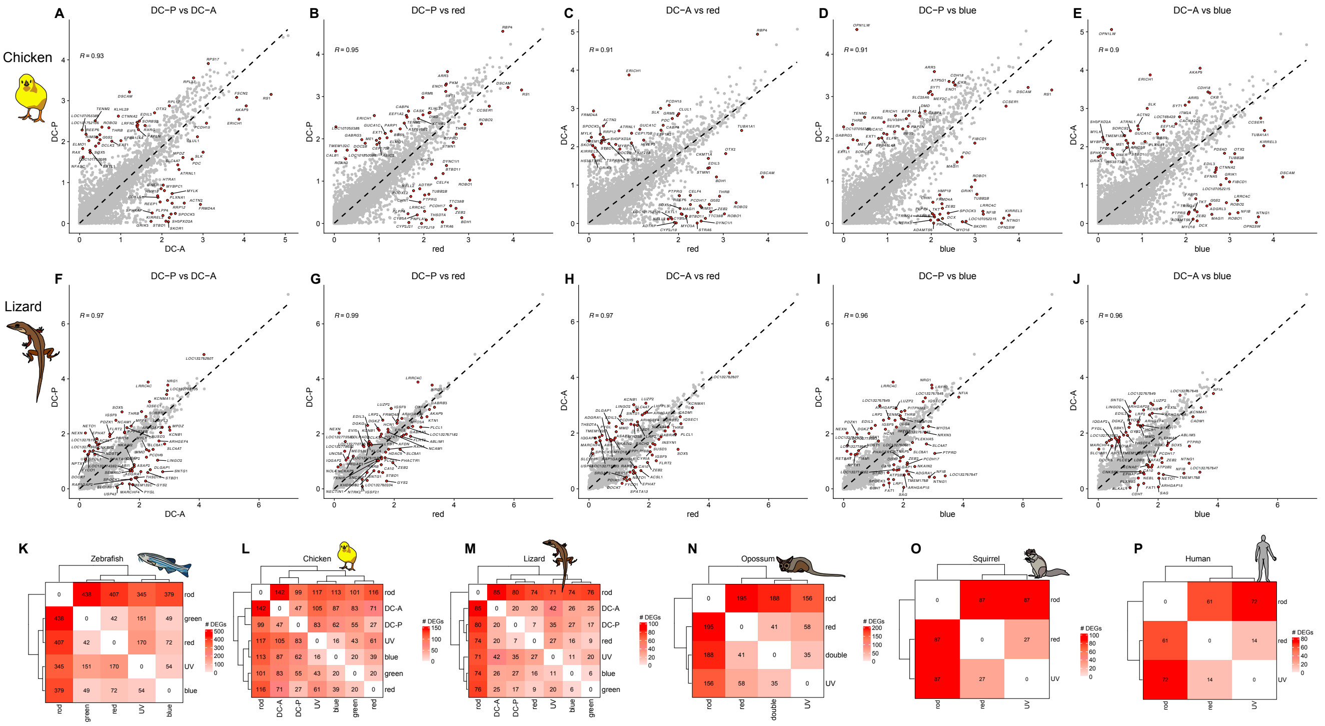

Figure S5: Genes that distinguish DC-P from DC-A, and DC-P or DC-A from red and blue cones, related to Figure 5. Scatterplots comparing normalized and log-transformed gene counts between two selected PR clusters in chicken (A-E) and lizard (F-J). Chicken: A) DC-P vs DC-A. B) DC-P vs red. C) DC-A vs red. D) DC-P vs blue. E) DC-A vs blue. Lizard: F) DC-P vs DC-A. G) DC-P vs red. H) DC-A vs red. I) DC-P vs blue. J) DC-A vs blue. Top 30 differentially expressed genes (DEGs) are labeled – a full list is provided in **Table S2**. B) Heatmaps showing number of pairwise differentially expressed genes (Benjamini-Hochberg-adjusted $p < 0.001$ and $\log_2$ fold change change > 1) between clusters for each species. Hierarchical clustering was performed using complete linkage and the number of DEGs as the distance.

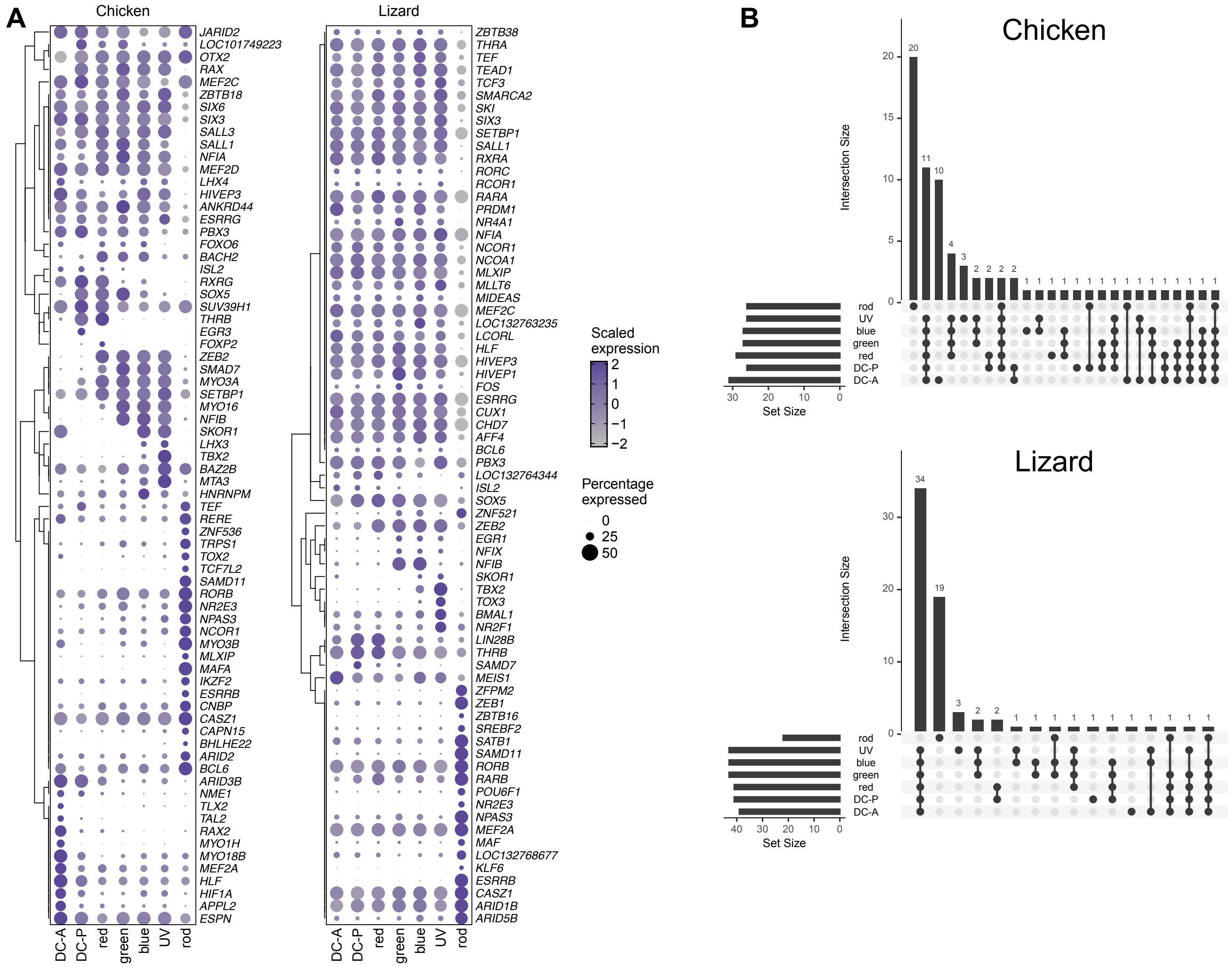

Figure S6: Analysis of transcription factor (TF) patterns in chicken and lizard photoreceptor types, related to Figure 5. A) Dot plot showing scaled expression values as the color and percentage of cells with expression as the size. *Left panel:* Chicken TFs expressed in at least 15% of cells in at least one cluster. TFs are sorted by expression pattern. *Right panel:* Lizard TFs expressed in at least 15% of cells in one cluster. TFs are sorted by pattern. The hierarchical clustering tree for the genes was constructed from the binarized expression matrix using Manhattan distances and complete linkage. B) Upset plots[S5] showing the size of the various TF patterns observed in **A** Chicken TFs (top) and Lizard TFs (bottom).

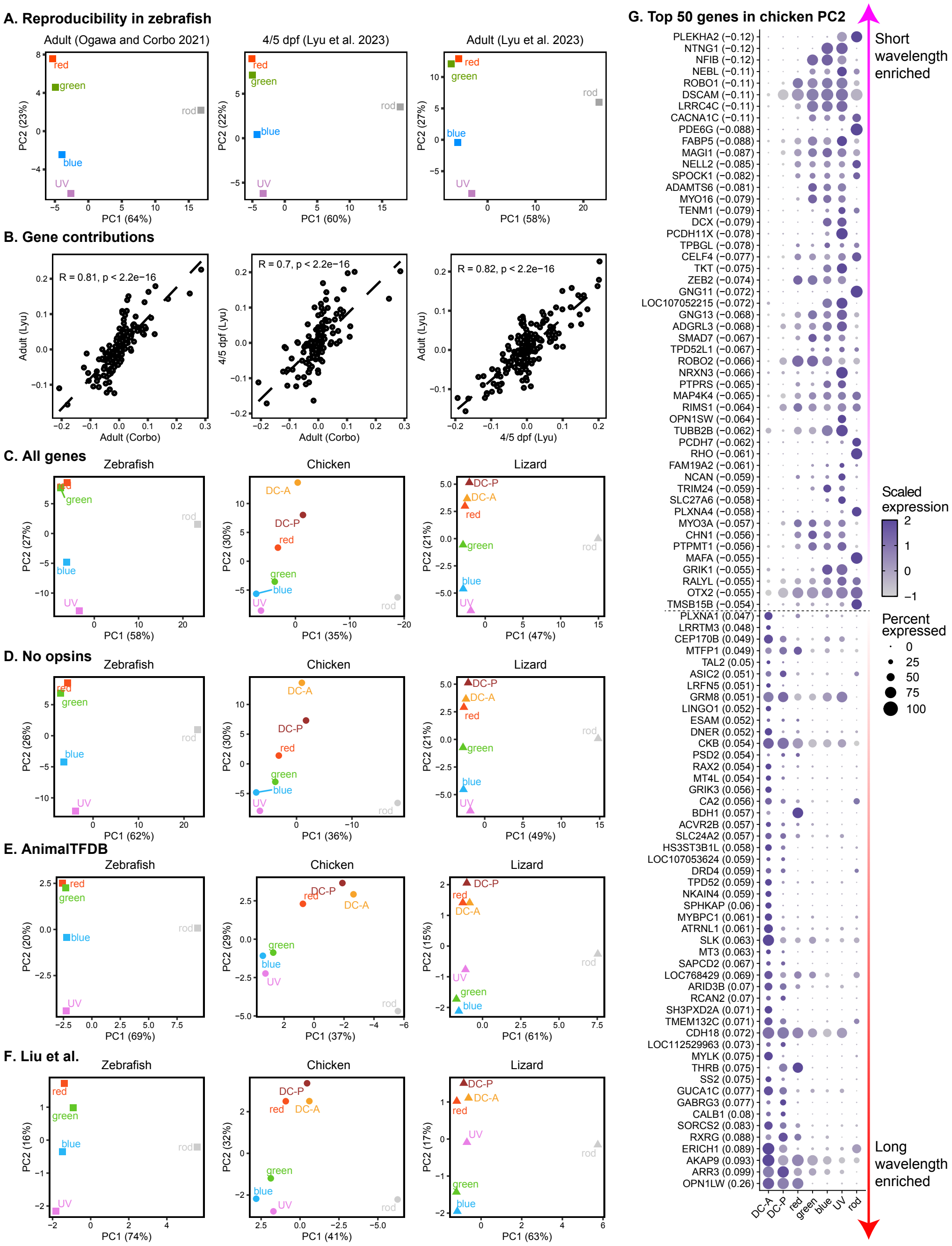

Figure S7: Robustness of principal component 2 in various datasets and conditions, related to Figure 6. A) PCA of averaged photoreceptor gene expression profiles within three zebrafish datasets (columns)[S6,S7]. In all three cases, $PC1$ separates rods and cones, while $PC2$ separates cone types based on their color. B) Scatterplots comparing $PC2$ gene contributions (loadings) between pairs of datasets in panel **A**. C-F) Principal component analysis (PCA) of zebrafish, chicken, and lizard photoreceptor gene expression with different gene sets: C) PCA with all genes (exactly like **Figure 6A**). D) PCA with all genes minus visual opsins. E) Using only ∼1500 TFs from AnimalTFDB 3.0. F) Using only ∼100 highly variable TFs from Liu et al.[S4]. G) Dot plot showing the top 50 positive and top 50 negative gene contributors to PC2 from chicken in panel **C**. Color shows average scaled expression while dot size shows the percentage of cells with expression. Value shown in parentheses next to gene shows the PC loading value (contribution to the PC).

Supplemental References

S1. Yamagata, M., Yan, W., and Sanes, J. R. (2021). A cell atlas of the chick retina based on single-cell transcriptomics. eLife *10*, e63907. https://doi.org/10.7554/eLife.63907.

S2. Kram, Y. A., Mantey, S., and Corbo, J. C. (2010). Avian Cone Photoreceptors Tile the Retina as Five Independent, Self-Organizing Mosaics. PLoS ONE *5*. Ed. by E. Warrant, e8992. https://doi.org/10.1371/journal.pone.0008992.

S3. Hu, H., Miao, Y., Jia, L., Yu, Q., Zhang, Q., and Guo, A. (2019). AnimalTFDB 3.0: a comprehensive resource for annotation and prediction of animal transcription factors. Nucleic Acids Res. *47*, D33–D38. https://doi.org/10.1093/nar/gky822.

S4. Liu, Y., Hurley, E. C., Ogawa, Y., Gause, M., Toomey, M. B., Myers, C. A., and Corbo, C. J. (2025). Avian photoreceptor homologies and the origin of double cones. Curr. Biol. *35*, XXX–XXX.

S5. Conway, J., Lex, A., and Gehlenborg, N. (2017). UpSetR: an R package for the visualization of intersecting sets and their properties. Bioinformatics *33*, 2938–2940. https://doi.org/10.1093/bioinformatics/btx364.

S6. Ogawa, Y. and Corbo, J. C. (2021). Partitioning of gene expression among zebrafish photoreceptor subtypes. Sci. Rep. *11*, 17340. https://doi.org/10.1038/s41598-021-96837-z.

S7. Lyu, P., Iribarne, M., Serjanov, D., Zhai, Y., Hoang, T., Campbell, L., Boyd, P., Palazzo, I., Nagashima, M., Silva, N., et al. (2023). Common and divergent gene regulatory networks control injury-induced and developmental neurogenesis in zebrafish retina. Nat. Commun. *14*, 8477. https://doi.org/10.1038/s41467-023-44142-w.
